# Native mass spectrometry of human carbonic anhydrase I and its inhibitor complexes

**DOI:** 10.1007/s00775-020-01818-8

**Published:** 2020-09-14

**Authors:** Carlotta Zoppi, Alessio Nocentini, Claudiu T. Supuran, Alessandro Pratesi, Luigi Messori

**Affiliations:** 1 Laboratory of Metals in Medicine (MetMed), Department of Chemistry “Ugo Schiff”, University of Florence, Via della Lastruccia 3-13, 50019 Sesto Fiorentino, Italy; 2 Department of Neurofarba, Section of Pharmaceutical and Nutraceutical Sciences, University of Florence, Via U. Schiff 6, 50019 Sesto Fiorentino, Italy; 3 Department of Chemistry and Industrial Chemistry, University of Pisa, Via G. Moruzzi 13, 56124 Pisa, Italy

**Keywords:** Human carbonic anhydrase, Mass spectrometry, Carbonic anhydrase inhibitor, Native protein analysis

## Abstract

**Abstract:**

Native mass spectrometry is a potent technique to study and characterize biomacromolecules in their native state. Here, we have applied this method to explore the solution chemistry of human carbonic anhydrase I (hCA I) and its interactions with four different inhibitors, namely three sulfonamide inhibitors (AAZ, MZA, SLC-0111) and the dithiocarbamate derivative of morpholine (DTC). Through high-resolution ESI-Q-TOF measurements, the native state of hCA I and the binding of the above inhibitors were characterized in the molecular detail. Native mass spectrometry was also exploited to assess the direct competition in solution among the various inhibitors in relation to their affinity constants. Additional studies were conducted on the interaction of hCA I with the metallodrug auranofin, under various solution and instrumental conditions. Auranofin is a selective reagent for solvent-accessible free cysteine residues, and its reactivity was analyzed also in the presence of CA inhibitors. Overall, our investigation reveals that native mass spectrometry represents an excellent tool to characterize the solution behavior of carbonic anhydrase.

**Graphic abstract:**

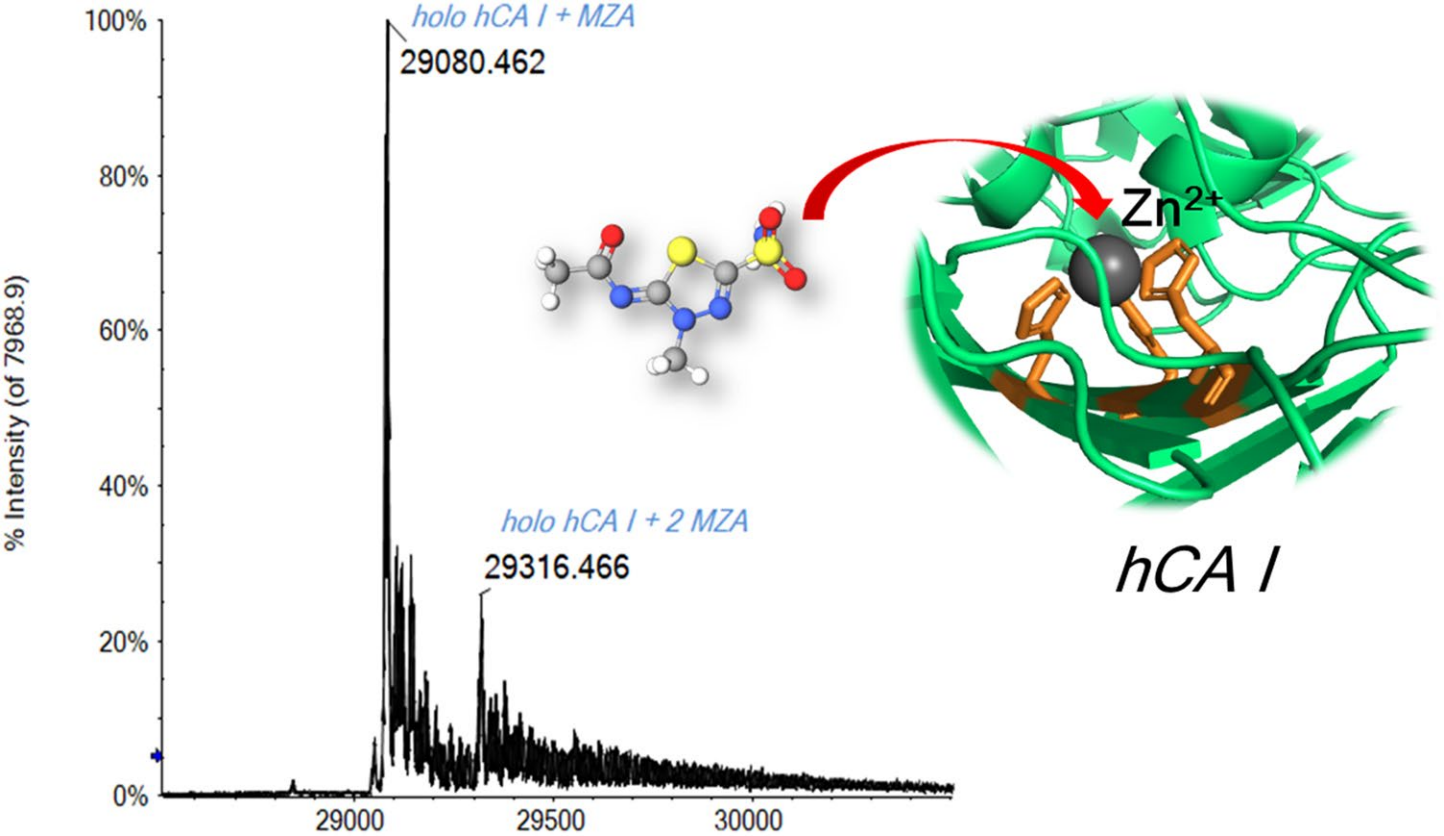

**Electronic supplementary material:**

The online version of this article (10.1007/s00775-020-01818-8) contains supplementary material, which is available to authorized users.

## Introduction

Carbonic anhydrase (CA, EC 4.2.1.1) is a zinc metalloenzyme that catalyzes the reversible hydration of carbon dioxide to bicarbonate according to the following equation: CO_2_ + H_2_O ⇆ HCO_3_^−^ + H^+^ [1, 2]. The HCO_3_^−^/CO_2_ equilibrium is critical for human health, and its inhibition has been a goal of therapeutic intervention for several decades [3–6]. The majority of known CA inhibitors contain a primary sulfonamide group [7, 8]. The sulfonamide anion, RSO_2_NH^−^, coordinates to the active-site zinc and may form hydrogen bonds with active-site amino acid residues located in the immediate vicinity further stabilizing the enzyme/inhibitor complex [9].

Owing to the importance and variety of therapeutic applications, the search for inhibitors of carbonic anhydrase is still very intensely pursued. This search is greatly assisted by a precise knowledge of the binding mode of the inhibitors to the enzyme at the atomic level. To this end, a large number of biophysical methods including NMR, X-ray crystallography, surface plasmon resonance (SPR), etc. have been exploited during the last few decades. A detailed description of these methods and of the information that can be derived may be found in a few reviews [10–12].

Among the various biophysical techniques, mass spectrometry turned out to be a potent method to describe drug–target interactions [13–16]. Specifically, native mass spectrometry is a powerful technique to study and characterize biomolecules in their native state, as the gentle ionization method preserves in the gas phase the supramolecular interactions, the conformational features, and the non-covalent association with ligands that are present in solution. In native MS, a strict control of pH, temperature, presence of non-denaturing co-solvents, and of the instrumental parameters is required to guarantee not only the retention of the tertiary structure of the biomolecule, but also the biological function, in the case of an enzyme the catalytic activity [17, 18]. Native MS started in the 90s’ with the pioneering works of Ganem and Katta, who first independently demonstrated that non-covalent interactions between biomolecules can be preserved and transferred from solution to the gas phase, allowing their detection via ESI–MS. Ganem et al. successfully detected via ESI–MS the complex between the receptor FKBP and its ligand, the macrolide FK506 [19]. Katta et al*.* reported that the non-covalent heme–globin complex of myoglobin is preserved in the gas phase generated via electrospray [20]. Since then, ESI–MS has been recognized as an election tool to investigate and characterize many ligand–biomolecule interactions: protein–cofactors, protein–DNA, protein–metal–drug, enzyme–substrate, enzyme–inhibitors, and antigen–antibody, and a plethora of papers and reviews has been published about this issue [16, 21, 22].

Native-MS has many analytical advantages. The identification of the fragment bound to the biomolecule and the binding stoichiometry can be directly inferred simply from the inspection of the mass spectrum. In fact, once detected the target biomolecule signal, any shift toward greater mass values is a sign of the binding with a ligand whose mass is equal to the mass shift detected. Moreover, the high sensitivity of mass spectrometry requires just a very small sample quantity for analysis, a few micrograms compared to the larger quantities required by other methods, such as NMR and crystallography. These significant features make native-MS a compelling screening method for the fragment-based drug discovery (FBDD) allowing the identification of chemotypes that bind to a protein, even through weak interactions [13]. In a recent work of Woods et al., native MS has been successfully applied in a fragment screening analysis toward CA II, to disclose new potential inhibitors of the enzyme [23]. Native MS has been successfully proven to be a valid alternative to the traditional screening methods, such as SPR and X-ray crystallography, offering unique advantages over them, as no sample manipulation and a very small sample concentration are required.

Another significant biological application of native-MS concerns the structural investigation of proteins. Certainly, since the pioneering work of Chowdhury et al*.* in 1990, where the conformational changes of Cytochrome c have been monitored for the first time by ESI–MS, the ability of the native-MS to probe and characterize the conformational state of proteins is well known [24].

Indeed, during the soft ionization process occurring in the ESI source, the protein can take multiple charges in accordance with how many protonable (or deprotonable) residues it exposes to the source, giving rise to multicharged species’ signals in the mass/charge spectrum. This signals ensemble represents the charge state distribution (CSD) of the protein. The average charge state that a protein takes on depends on its tertiary structure and its solvent-accessible surface area: the more the residues are buried in the structure, fewer charges the protein can take [25, 26]. Among the many factors that influence the CDS (i.e., solvent, parameters of the instrument, etc.), the protein conformation is the most important [27, 28]. Indeed, it is widely documented that the unfolding of a protein in denaturing conditions causes the shift of its CDS toward higher charges (low *m/z* values) due to a greater accessibility of basic or acidic residues that can accommodate charges [28, 29]. In a study of Nabuchi et al*.*, the unfolding and refolding processes of CA triggered via pH modulation were monitored by ESI mass spectrometry. These authors followed the conformational changes through the monitoring of the mass shift associated with zinc release in the unfolded state, and through the variation of the charge-state distribution of the protein signal [30].

**Scheme 1:**
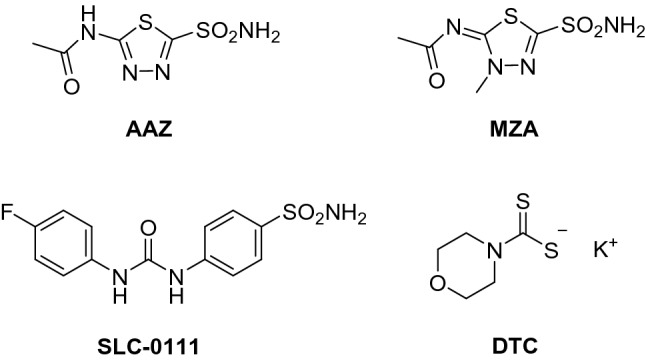
Chemical structures of the selected hCA I inhibitors: acetazolamide (AAZ), methazolamide (MZA), SLC-0111, and potassium morpholine-4-carbodithioate (DTC)

In this manuscript, we exploit native mass spectrometry to characterize the solution chemistry of hCA I and investigate its interactions with a few selected inhibitors. Specifically, four distinct inhibitors were chosen (Scheme 1) of which three are sulfonamide inhibitors, namely acetazolamide (AAZ), methazolamide (MZA), and SLC-0111, and the fourth one is a dithiocarbamate (potassium morpholine-4-carbodithioate, DTC). All these four inhibitors are known to produce their effect through direct binding to the Zn(II) ion, as the X-ray crystal structures of their adducts with various CAs have been reported [31–34]. AAZ and MZA are the first-generation CAIs used as systemic drugs for the management of glaucoma and as standard CAIs in many pharmacological investigations [5]. SLC-0111 is the first-in-class selective CA IX/XII inhibitor progressing to clinical trials [32]. It successfully completed and passed Phase I, and entered in Phase Ib/II clinical trials in 2017 for the treatment of advanced, metastatic solid tumors [35]. DTC is a main representative of dithiocarbamates, a class of potent zinc-binder CAIs, second only to sulfonamide-like derivatives [31]. In Table 1, the hCA I inhibition constants of the four inhibitors are reported. In addition, further studies are conducted concerning the reaction of hCA and its inhibitor complexes with the gold(I) drug auranofin that is known to bind selectively the free cysteine residue (Cys212). Overall, a quite detailed and satisfactory description of the occurring processes is achieved.

**Table 1:** hCA I inhibition constants

hCA I inhibitor	*K*_I_ (nM)*
AAZ	250
MZA	50
SLC-0111	5080
DTC	0.88

*The reported *K*_I_ values were previously published in refs. [5, 31]

## Materials and methods

### Materials

Lyophilized human carbonic anhydrase (hCA I) was purchased from Sigma-Aldrich and used without further purification or manipulation. Sulfonamide inhibitors of hCA I were synthetized by one of our groups [31, 32] or are commercially available from Sigma-Aldrich (Milan, Italy). Auranofin (AF) was purchased from Enzo Life Sciences (Farmingdale, New York). Water, methanol, and ammonium acetate were of LC–MS grade and were purchased from Sigma-Aldrich.

### Sample preparation

The stock solution of hCA I 10^–4^ M was prepared dissolving the protein in H_2_O LC–MS grade. Stock solutions (10^–2^ M) of the inhibitors were prepared dissolving the samples in DMSO. AF was freshly prepared in LC–MS grade water and methanol (50:50 v/v) to a final concentration of 3 × 10^–3^ M.

For the ESI–MS experiments with hCA I, aliquots of the protein stock solution were diluted to 7 × 10^–7^ M with ammonium acetate solution 2 × 10^–3^ M, pH 6.8.

For the experiments with the inhibitors, solutions of hCA I 10^–5^ M and each inhibitor at fixed protein-to-inhibitor ratio (1:1, 1:3, 1:10) were prepared diluting with ammonium acetate buffer 2 × 10^–3^ M, pH 6.8. The mixtures were then incubated at 37 °C for 5 min.

For the experiment with AF, a solution of hCA I 10^–5^ M and AF at 1:3 protein-to-metal complex ratio was prepared and diluted with ammonium acetate solution 2 × 10^–3^ M, pH 6.8. The solutions were then incubated at 37 °C for 2 h and then diluted to a final protein concentration of 7 × 10^–7^ M using ammonium acetate solution 2 × 10^–3^ M, pH 6.8.

### ESI–MS analysis: final dilutions

After the incubation time, all solutions were sampled and diluted to a final protein concentration of 7 × 10^–7^ M using ammonium acetate solution 2 × 10^–3^ M, pH 6.8

In the non-native-like experiments, the final solutions were also added with 0.1% v/v of formic acid just before the infusion in the mass spectrometer.

### Instrumental parameters

The ESI mass study was performed using a TripleTOF^®^ 5600^+^ high-resolution mass spectrometer (Sciex, Framingham, MA, USA), equipped with a DuoSpray^®^ interface operating with an ESI probe. Respective ESI mass spectra were acquired through a direct infusion at 5 μL/min flow rate.

The general ESI source parameters optimized for hCA I analysis were as follows:

- Positive polarity, Ionspray Voltage Floating 5500 V, Temperature 0, Ion source Gas 1 (GS1) 40 L/min; Ion source Gas 2 (GS2) 0; Curtain Gas (CUR) 10 L/min, and Collision Energy (CE) 10 V.
- Selective variations of some parameters were applied for DP value adjustment: for native hCA I and milder DP, a value of 200 V was used and 2500–5000 *m/z* mass range; for native hCA I and harder DP, a value of 300 V was used and 2500–5000 *m/z* mass range; for denatured hCA I positive polarity, Ionspray Voltage Floating 5500 V, Temperature 0, Ion source Gas 1 (GS1) 50 L/min; Ion source Gas 2 (GS2) 0; Curtain Gas (CUR) 20 L/min, Declustering Potential (DP) 50 V, Collision Energy (CE) 10 V, range 760–990 *m/z*.
- For acquisition, Analyst TF software 1.7.1 (Sciex) was used and deconvoluted spectra were obtained by using the Bio Tool Kit micro-application v.2.2 embedded in PeakView™ software v.2.2 (Sciex).

## Results and discussion

### Native-MS analysis of hCA I

Before investigating the protein-binding properties of the selected inhibitors, it was necessary to assess the best conditions for the ESI–MS experiment to observe the protein in its native-like state. In accordance with the definition of “native mass spectrometry” proposed by Heck [17], we aimed to preserve the protein tertiary structure and the binding of the Zn ion in the enzyme active site, as most inhibitors like sulfonamides and dithiocarbamates directly bind this metal ion [36]. The Zn ion is located in a cone-shaped pocket and is coordinated to three His residues and a water molecule (or OH^−^ ion) in a roughly tetrahedral geometry. The His residues are invariant in the whole α-CA family: they are His94, His96, and His119. Other neighboring residues complete the coordination shell establishing hydrogen bonds [37].

**Fig. 1:**
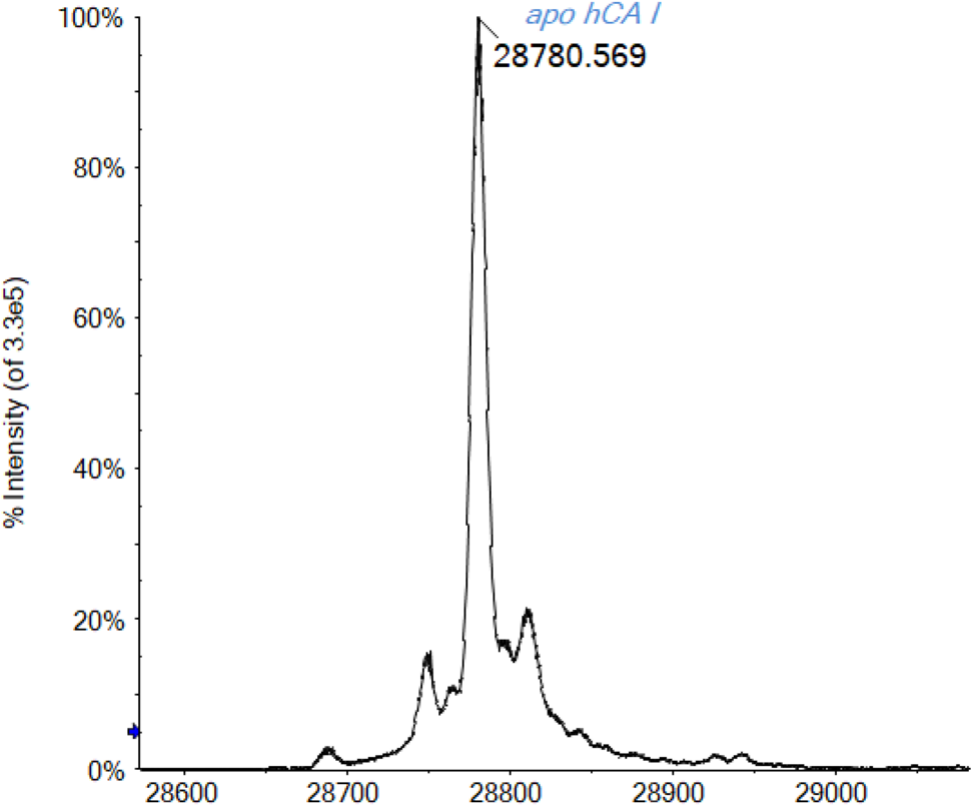
Deconvoluted ESI-Q-TOF mass spectrum of hCA I 7 × 10^–7^ M in ammonium acetate solution 2 × 10^–3^ M (pH 6.8). 0.1% v/v of formic acid was added prior to infusion

In a recent work of ours, CA I was investigated in depth through an established protocol of protein ESI–MS analysis [38]. This methodology includes the direct injection into the ESI-Q-TOF mass spectrometer of a protein sample dissolved in ammonium-acetate solution (see “Sample preparation”). Normally, a small percentage (0.1% v/v) of formic acid is added just before the injection into the mass spectrometer, to enhance the ionization process in the ESI source. Unfortunately, under these experimental conditions, the ESI–MS spectrum shown in Fig. 1 reveals that only apo-CA I is detectable. Notably, the signal at 28,780 Da is assigned to the apo protein and corresponds to the molecular weight of the hCA I amino acid sequence (Uniprot P00915) with the loss of Met1 and the presence of one acetylation on Ala2 residue.

The addition of 0.1% v/v of formic acid to the ammonium acetate solution induces a lowering of the pH from 6.8 to 2.9 with the rapid release of the zinc ion from the protein. Indeed, the His residues that coordinate the metal ion in the active site of the enzyme possess a p*K*a < 6, and in an acidic solution, they may undergo facile protonation, causing the loss of the zinc ion [39–42]. As a confirmation, the study of Coleman demonstrated, through optical rotatory dispersion, that hCA B (the old name for hCA I) loses irreversibly its native conformation under pH 4, with the consequent release of the Zn ion and the loss of the catalytic activity [43].

Most of the proteins analyzed in our previous experience with ESI–MS retained their native conformation under physiological-like conditions (i.e., with ammonium acetate solution, ammonium hydrogen carbonate, or water with the addition of 0.1% v/v of formic acid for the mass spectrometry analysis), as proved by our extensive works published during the last years [38, 44–49]. However, the present case can be extremely instructive given the particular sensitivity of hCA I for acidic conditions. It is noteworthy to remind that the so-called “native conditions” for mass spectrometry analysis of proteins in their biologically active conformation cannot be generalized in a standard method, but rather carefully set for each protein [17, 50, 51].

**Fig. 2:**
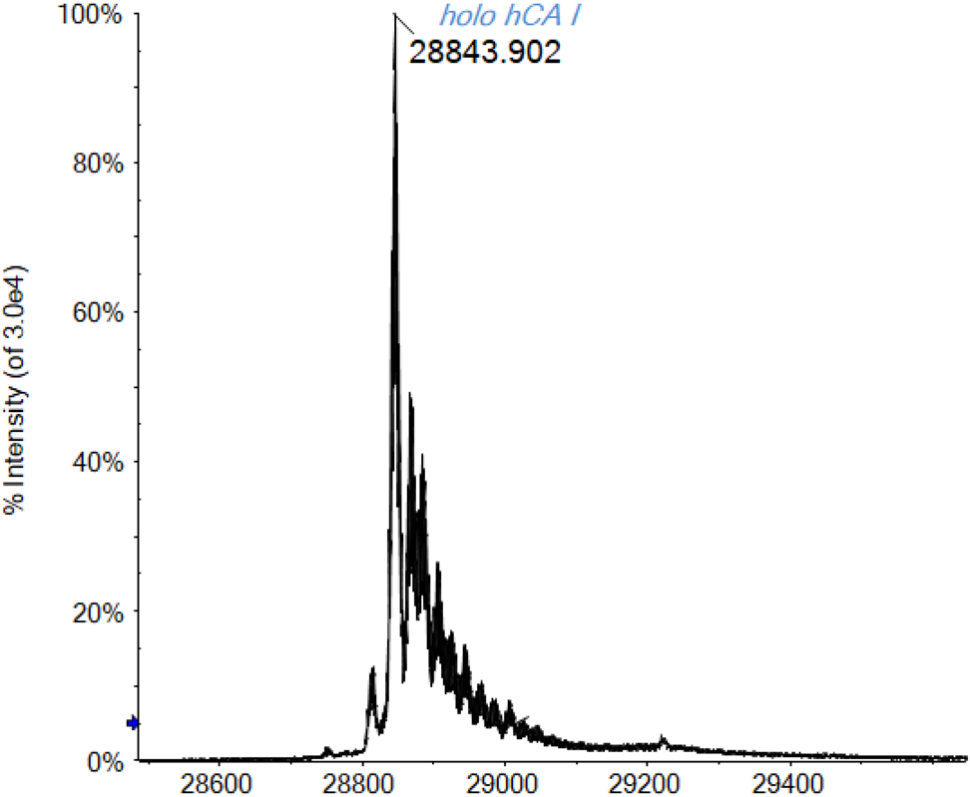
Deconvoluted ESI-Q-TOF mass spectrum of hCA I 7 × 10^–7^ M in ammonium acetate solution 2 × 10^–3^ M (pH 6.8)

For this reason, the first experiments were devoted to the identification of the best experimental conditions to avoid protein unfolding and denaturation, besides the search of the best ionization performances. First of all, in the case of hCA I, the absence of acid in the ammonium acetate solution is one of the key point for ESI–MS experiments in native-like conditions. Moreover, in this case, also an adequate ionization is reached, leading to a well-resolved spectrum of holo-hCA I displayed in Fig. 2. The main signal at 28,843 Da is attributable to holo hCA I with the Zn(II) ion retained in its coordination site. Besides, signals of protein adducts with sodium (+ 23 Da) and oxidized forms of the protein (+ 16 Da) are also observed.

Therefore, non-denaturing conditions preserve the zinc binding to the active site of the enzyme, while the acidification induces the release of the metal center [52, 53].

In this regard, by comparison between the multicharged spectra of hCA I obtained in acidic and neutral conditions, some interesting observations can be proposed about the different protein conformation.

**Fig. 3:**
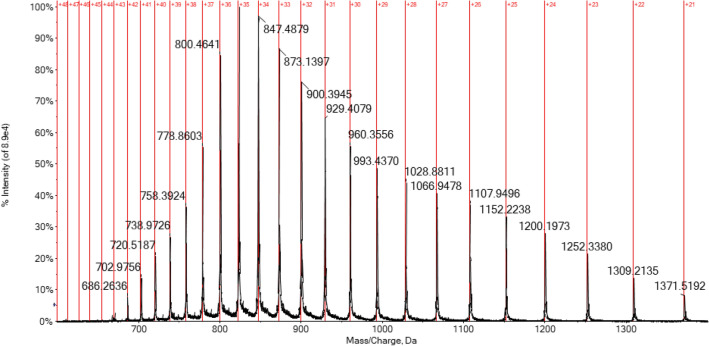
CDS in ESI-Q-TOF multicharged spectrum of hCA I 7 × 10^–7^ M in ammonium acetate solution 2 × 10^–3^ M (pH 6.8) and 0.1% v/v of formic acid

Since folded protein molecules can accommodate fewer charges in comparison to the unfolded counterpart, the analysis of the charge-state distribution (CSD) in the multicharged spectra can give some clear information on the conformational state of the protein [28]. A drastic variation of CDS in the spectrum of CA, has been highlighted among both experimental conditions. Reasonably, the acidic conditions also cause a considerable alteration of the protein tertiary structure, giving rise to a partial unfolding and, thus, to a higher degree of protonation in the ESI source [54–56]. As a result, with the addition of formic acid, the CDS becomes broader and shifted to highly charged ions, with a maximum at + 35 protonation state, as depicted in Fig. 3.

**Fig. 4:**
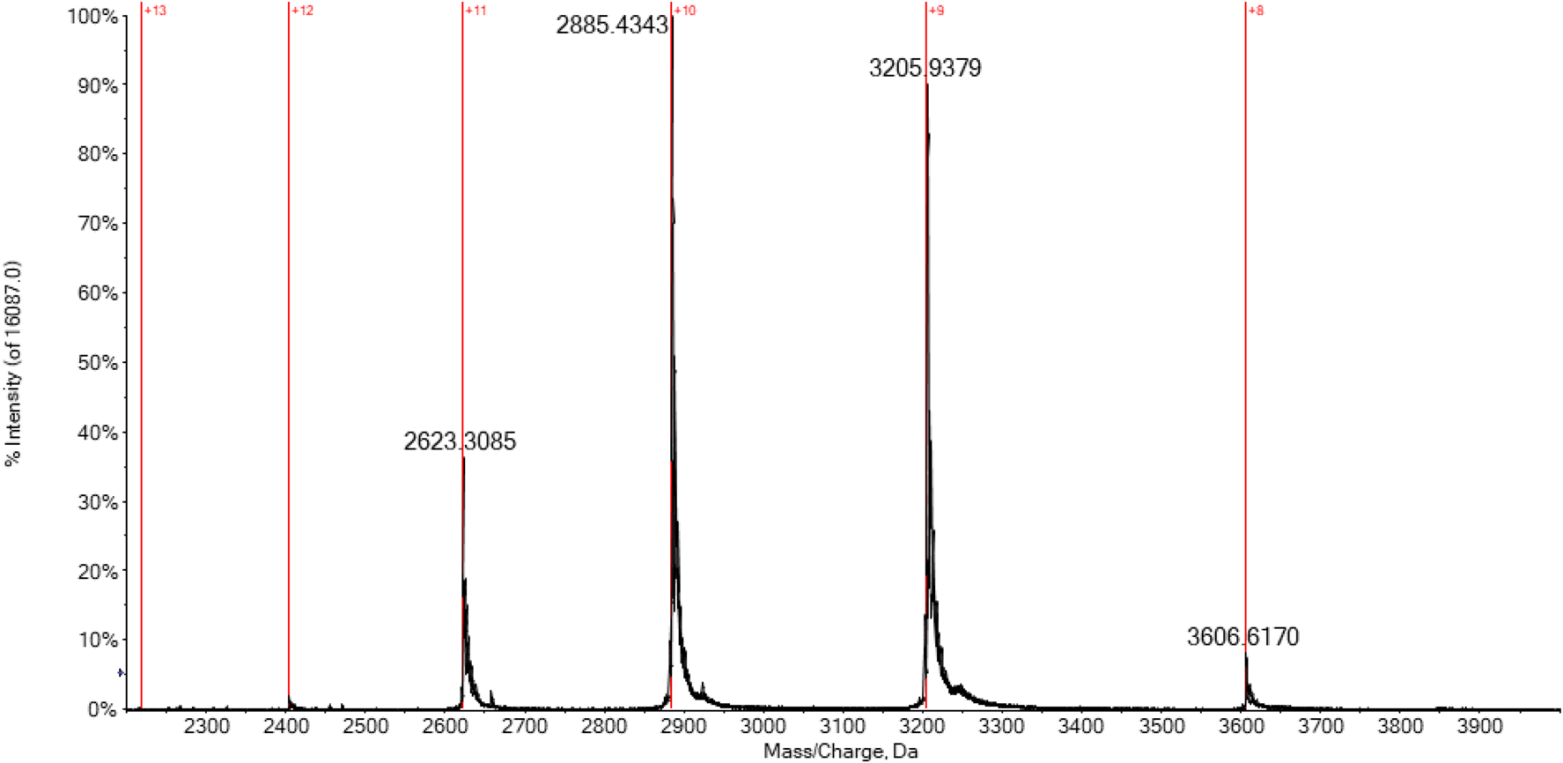
CDS in ESI-Q-TOF multicharged spectrum of hCA I 7 × 10^–7^ M in ammonium acetate solution 2 × 10^–3^ M (pH 6.8)

Contrariwise, the spectrum in Fig. 4 has been performed on the CA solution in non-acidic conditions and shows a narrower CDS with a maximum charge state of + 10. Therefore, it is evident that the acidification induces a partial loss of the tertiary structure of the protein, exposing more amino acid residues to the solvent and then leading to a larger protonation in the electrospray compared to the folded state [27].

Another important consideration that may help to better characterize the protein and its interactions via mass spectrometry is related to the greater instrumental resolution achievable when dealing with lower CDS spectra. In fact, a lower charge state corresponds to a greater separation (being ∆*m* = 1/*z*) between consecutive signals in the multicharged isotopic cluster increasing, in case of a Tof instrument, the FWHM resolution [57–59]. As a result, the mass spectrum of CA in native-like conditions reported in Fig. 2 is more isotopically resolved and informative with respect to the spectrum in Fig. 1 recorded for a solution of CA under slightly acidic conditions (0.1% v/v formic acid).

### The binding of the inhibitors

As reported in the literature, sulfonamide and dithiocarbamate inhibitors bind directly to the Zn ion of the CA [36]. Through high-resolution ESI–MS analysis, we succeeded in providing a clear demonstration of such a binding. Indeed, the interaction between hCA and a small panel of its inhibitors (Scheme 1) can be investigated in detail and the adducts formed can be easily observed just in native conditions, in which the Zn ion binding to the three histidine residues in the active site of the enzyme is preserved.

First, we investigated the adduct formation of CA with the inhibitors in native-like conditions, varying properly the instrumental parameters to optimize the adduct signal.

**Fig. 5:**
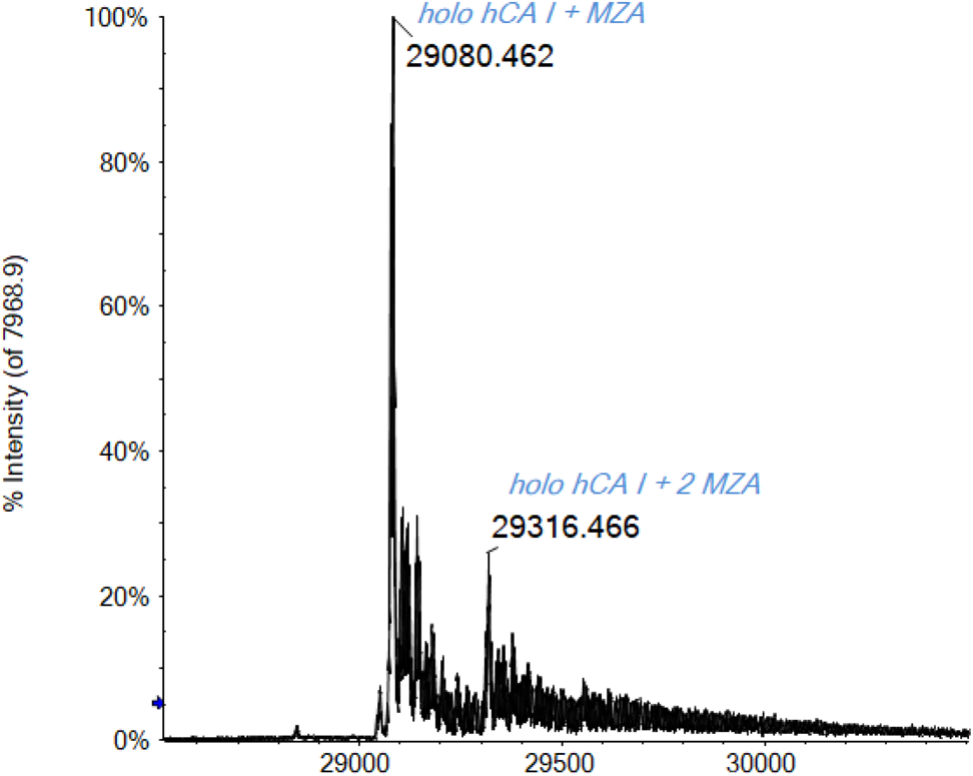
Deconvoluted ESI-Q-TOF mass spectrum of hCA I 7 × 10^–7^ M incubated for 5 min at 37 °C with MZA (1:10 protein/inhibitor ratio) in ammonium acetate solution 2 × 10^–3^ M (pH 6.8), DP 200 V

Different aliquots of a 10^–5^ M protein solution were prepared in the presence of the selected inhibitor (at 1:10 protein-to-inhibitor ratio) in ammonium acetate solution. Each mixture was incubated at 37 °C for five minutes and then diluted to the final concentration with the same ammonium acetate solution (pH 6.8). The ESI–MS spectra were then acquired, and adduct formation was assessed for each of the four inhibitors. From the literature, it is known that the sulfonamide inhibitors bind the Zn ion through their R-SO_2_-NH^−^ moiety [9, 60, 61], while the dithiocarbamate through the R_2_NCS_2_^−^ moiety [31, 62]. For example, Fig. 5 reports the deconvoluted mass spectrum of MZA incubated with hCA I.

Notably, the unbound protein signal at 28,843 Da is no longer observed, indicating that CA reacts completely with the inhibitor. The main signal detected at 29,080 Da perfectly matches the mass of the MZA/CA adduct formed through the binding of the R-SO_2_-NH^−^ moiety to the Zn ion. Moreover, a signal corresponding to a bis adduct is also detected at 29,316 Da with a relative intensity of about 20% respect to the main peak. The formation of bis adducts between sulfonamide inhibitors and CA, although apparently surprising, is in perfect agreement with some NMR and MS studies already reported in the literature [63, 64]. In fact, as described by Whitesides and co-workers, CA retains the tertiary structure of its binding pocket in the gas phase on the time scale (seconds to minutes) of the ESI–MS measurements [63]. Therefore, although the binding stoichiometry between CA and its inhibitors was 1:1 in solution, these authors postulated that the second equivalent of inhibitor was likely condensed on the surface of the hCA I (and then at non-Zn(II) site) during the ESI desolvation process [63].

**Fig. 6:**
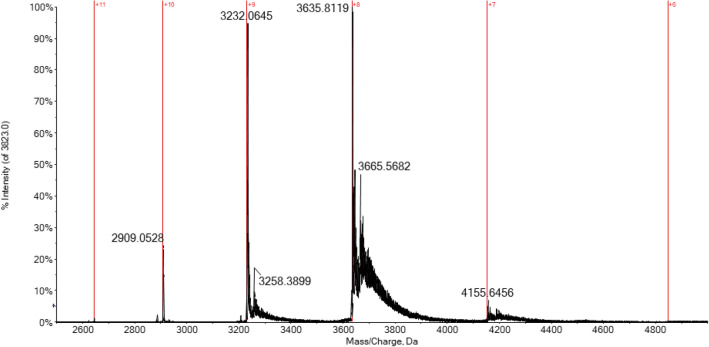
Multicharged ESI-Q-TOF mass spectrum of hCA I 7 × 10^–7^ M incubated with MZA (1:10 protein-to-inhibitor ratio) in ammonium acetate solution 2 × 10^–3^ M (pH 6.8) for 5 min at 37 °C

By comparing the multicharged spectrum of unreacted CA (Fig. 4) and that with a bound inhibitor, some conformational considerations can be proposed. As an example, in Fig. 6, the charge-state distribution for the CA/MZA adduct is reported. Interestingly, no relevant changes in the CDS can be observed in comparison to the unreacted protein. Indeed, the most abundant charge state shifts from + 10 to + 8, and no broadening of CDS can be observed. This experimental evidence leads us to reasonably exclude any significant variation of the overall protein conformation due to inhibitor binding [55]. Probably, the small variation in the CDS can be truly attributed to a slight and localized conformational variation of the enzyme binding pocket following the inhibitor binding. Likewise, the insertion of the inhibitor molecule inside the enzymatic pocket may be of hindrance to the amino acid protonation of pocket itself.

Nevertheless, when formic acid was later added to the mixture of the inhibitor and CA, the protein lost the Zn ion and, as expected, the inhibitor too. Indeed, in these slightly acidic conditions, only the apo-CA signal was still detectable, with the total disappearance of adduct-related peaks. To monitor this loss, we first prepared the solution of the enzyme with inhibitor in “native-like” condition as previously determined, and then we added the 0.1% v/v of formic acid just before the injection in the mass spectrometer. As a proof of concept, in the Supporting Information, the mass spectrum of CA reacted with another sulfonamide inhibitor, i.e., SLC-0111, is reported: the only signal detected at 28,780 Da refers to the apo-CA peak. Again, when the acid was added to the protein solution, the overall resolution rapidly dropped down and the relative CDS was at the higher values, confirming the pH-related behavior discussed in the previous paragraph.

Therefore, as we assessed from the comparison of the charge-state distribution of unreacted CA in both native and acidified condition, the acidification probably induces a partial loss of the protein tertiary structure making the protonation of the His residues in the active site possible and causing the release of the Zn(II) ion from the enzyme’s pocket [40, 65, 66]. On the contrary, the adduct between the enzyme and the inhibitor is clearly detectable in the absence of any acid addition, when the protein preserves its folded state, as clearly demonstrated from the CDS analysis.

Another important equilibrium that takes place upon varying the pH conditions is the protonation and deprotonation of the sulfonamide and dithiocarbamate reactive moieties. It has already been reported in the literature that the inhibitor binding was suppressed at low pH values (< 2.5), presumably due to neutralization of the negative charge on the zinc-reactive moiety [67].

Definitely, the ESI–MS data obtained for the hCA/inhibitor system perfectly agree with the knowledge on the CA behavior already gathered with other complementary techniques (i.e. XRD), pointing out that the inhibitor binds directly to the Zn ion, when the enzyme is in its native state with the Zn ion tightly bound to its active site [62].

### The declustering potential modulation: comparison between the Zn binding to the enzyme and the inhibitor binding to the Zn

Another experimental parameter that deeply influences the obtained results in native ESI–MS analysis of proteins, and particularly metalloproteins, is the declustering potential (DP) [68, 69]. Briefly, the DP is an electric potential difference applied between the orifice and the following lens of the mass spectrometer (in the region of rapid gas expansion from atmosphere into vacuum) [18]. This potential difference establishes an electric field accelerating the ions through the low-density gas and is commonly adjusted to provide the optimum signal-to-noise ratio for the compound of interest. In particular, protonated solvent clusters are collisionally stripped from clustered protein as the potential is increased. Again, low charge-state ions can be more effectively declustered, while high charge-state ions are normally fragmented. Since we demonstrated that the CDS is closely related to the solution pH, this latter parameter is not only responsible for the native conformation retention, but it also plays a pivotal role in the efficacy of the DP. Then, the control of DP is another fundamental instrumental parameter that can improve the instrumental resolution and, therefore, deserves to be carefully evaluated.

Some papers show that the DP can be modulated during the MS analysis, to probe the nature of protein–ligand association. The raising of DP can induce the dissociation of the weaker interactions between a protein and a ligand, while the covalent ones are retained [70, 71].

On the other hand, a high DP value can induce, yet, the dissociation of the weaker ligand–protein adducts [72]. Therefore, we started the systematic study of the interactions between hCA I and its inhibitors, once fixed the best pH conditions, varying the DP value until any significant variation in the multicharged spectrum (in terms of signals intensities, appearance/disappearance of signals at greater masses, resolution, and overall quality of the spectrum) is observed.

First, a solution of CA alone has been prepared and analyzed, as previously described, avoiding any acid addition. At low DP values (i.e., 100 V), as reported in Supporting Information, the signal of the holo-protein is followed by many signals of adducts with salts from the solution, typically observed in protein’s MS analysis, which considerably complicates the mass spectrum. Specifically, the very intense peak at 28,904 Da (shift = + 60 Da with respect to hCA signal) can be reasonably attributed to the addition of an acetate ion from the buffer solution, while the second very intense peak at 29,100 Da has not yet been clearly assigned but probably dues to some ionic clusters from the buffer solution that weakly interact with the protein surface.

By raising the DP value up to 300 V (see Fig. 2), those adducts with salts are destroyed and the mass spectrum becomes cleaner with the protein signal that emerges as the main one. Significantly, even with this high voltage applied, in this case, the Zn ion is retained on its binding site and no significant protein unfolding occurs. A clear and well-resolved holo-protein signal is then observed.

However, when the ESI–MS experiments have been performed with the CA and inhibitor mixed together in these latter conditions, completely different results have been gathered. Indeed, at 300 V, we observed a drastic reduction of intensity of the adducts signals. In the Supporting Information, are displayed the deconvoluted mass spectra for each of the four inhibitors. Notably, in all cases, the main signal is now the one of the unreacted holo-proteins at 28,843 Da. The intensity of the adduct signals drastically collapses at about 30–50% intensity compared to the unreacted protein signal. Interestingly, bis adduct signals are no longer detected. At the moment, we cannot provide a plausible explanation and this aspect will be the object of further investigations.

**Fig. 7:**
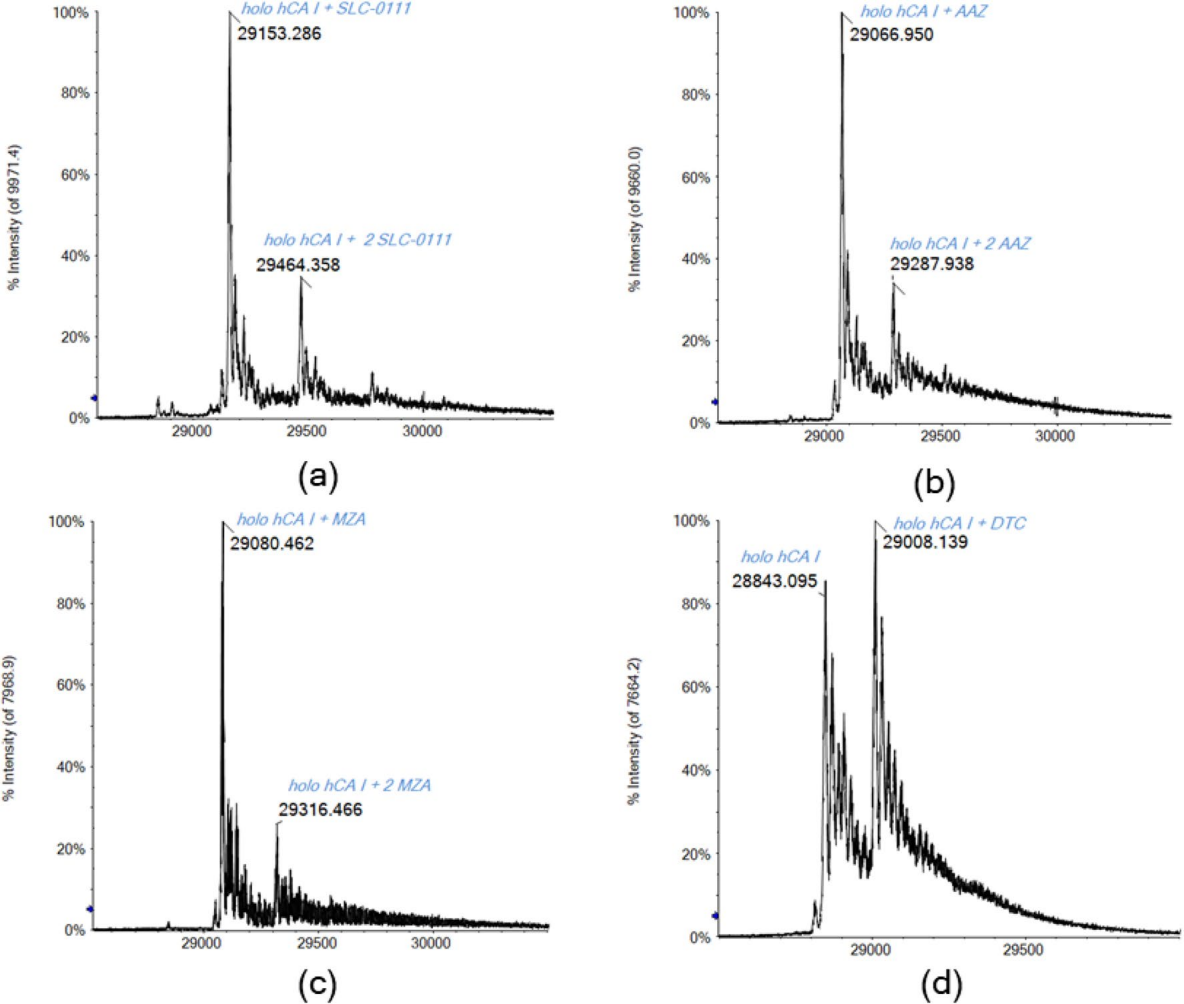
Deconvoluted ESI-Q-TOF mass spectrum of hCA I 7 × 10^–7^ M incubated for 5 min at 37 °C with MZA (**a**), SLC-0111 (**b**), AAZ (**c**), and DTC (**d**) (1:10 protein/inhibitor ratio) in ammonium acetate solution 2 × 10^–3^ M (pH 6.8), DP 200 V

Then, the DP value was decreased to the optimal value of 200 V and the recorded spectra are shown in Fig. 7. Now the peak corresponding to the unreacted protein is no longer detected in case of the three sulfonamides, but it is still present in case of DTC. Again, for the sulfonamide inhibitors, the main signal detected corresponds to the mono adducts with CA and further bis adducts are also revealed with a relative abundance of 30–40% respect to the first ones. A different behavior is observed for DTC; in this case, in addition to the partial adduct formation with CA, there is no trace about the bis adduct formation.

Thereafter, from the comparative analysis of the spectra obtained with different DP values, we can assert that 200 V can be considered the best value for ESI–MS analysis of CA/inhibitors adducts in native-like conditions, allowing to gather highly informative spectra with a clear and precise rendering of the protein reactivity towards those studied inhibitors.

Clearly, upon increasing the value of the applied voltage, the coordination bond between CA and the inhibitors is no longer preserved. Therefore, the observed residual mono-adduct signal at DP 300 V is due to the presence of a large excess of the inhibitor. Indeed, repeating the analysis on a solution with a 1:1 protein-to-inhibitor ratio, no adduct signal is observable for MZA, AAZ, and DTC, and only the protein signal is detected (see SI). The residual SLC-0111/CA adduct is probably due to the larger number of hydrogen bonds formed with other amino acid residues in the enzymatic pocket respect to the other inhibitors, making this adduct more resistant also to the higher DP values [9, 73].

Notably, although the DP set to the maximum value possible with our instrument (i.e., 300 V), it is clear that the protein preserves the Zn(II) ion into the active site, despite the high energy involved. Indeed, the Zn ion is tightly bound to the enzyme through various direct and indirect interactions with amino acid residues that stabilize the metal ion into the enzyme’s pocket [66].

Differently, the high DP values cause the fragmentation of the CA/inhibitors adducts with the loss of their respective signals. In this case, the kinetic energy applied to the system results too high to keep intact the inhibitors bound to the protein. Since the main signal in all the spectra at 300 V belongs to the holo-CA (see SI), this is suggestive for a different binding energy between Zn-protein and inhibitor-Zn adduct, resulting this latter one less stable with higher acceleration energy [74–76]. This is perfectly consistent with the great stabilization that the Zn ion receives from the coordination to the three histidine residues, while the inhibitor establishes only one bond with the metal ion [76].

### Competition between inhibitors

For a given inhibitor, the inhibition constant *K*_I_ represents the inhibitor concentration required to decrease the catalytic activity of the enzyme by 50%. Thus, *K*_I_ provides an estimate of the inhibitor’s affinity for the enzyme: more tightly the inhibitor binds to the enzyme active site, the smaller the amount of inhibitor required for inhibition [77].

To test the validity of this concept also with mass spectrometry, we carried out a competition experiment between two inhibitors with different *K*_I_. Specifically, the two sulfonamides AAZ and MZA were chosen, whose inhibition constants are *K*_I_ 250 nM and *K*_I_ 50 nM, respectively [78, 79]. Therefore, AAZ is our weaker inhibitor, while MZA is the stronger one. The protein was mixed with the two sulfonamide inhibitors in a 1:10:10 protein/inhibitor 1/inhibitor 2 ratio. We started the protein incubation with the less tight-binding inhibitor (AAZ), then we added the stronger one (MZA). After a further 5 min of incubation at 37 °C, the spectrum is acquired. Later, we also carried out the same experiment by reversing the order of inhibitor addition.

**Fig. 8:**
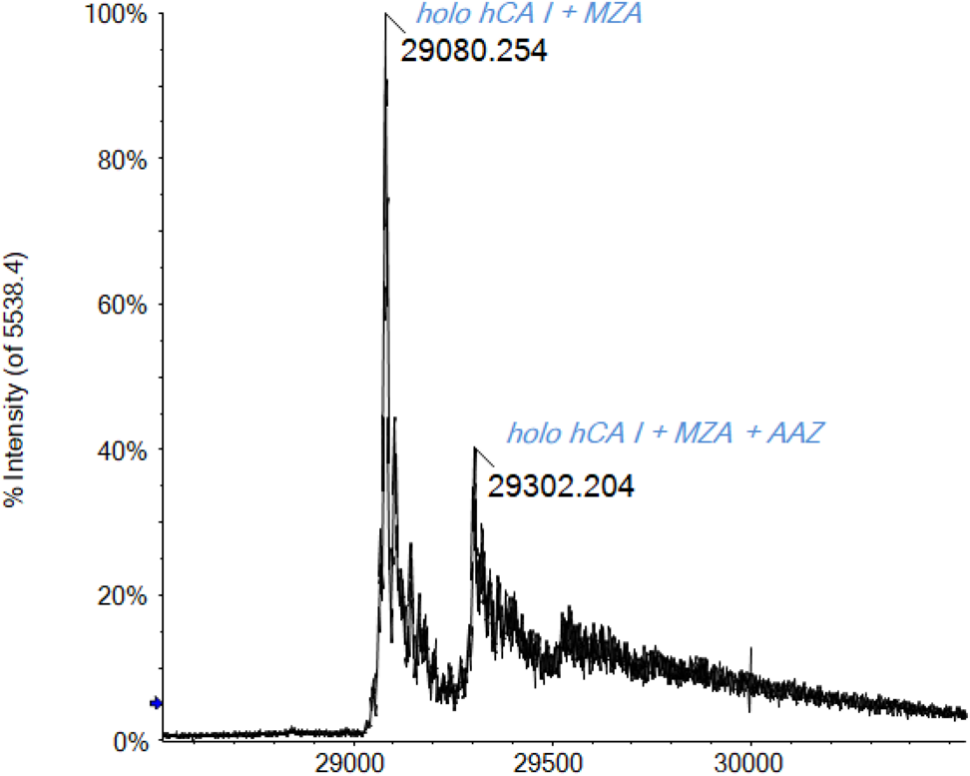
Deconvoluted ESI-Q-TOF mass spectrum of hCA I 7 × 10^–7^ M incubated for 5 min at 37 °C with AAZ and MZA (1:10:10 protein/inhibitor 1/inhibitor 2 ratio) in ammonium acetate solution 2 × 10^–3^ M (pH 6.8)

In the first experiment, AAZ is incubated with hCA I. The ESI mass spectrum (see SI) displays the intense signal of the CA/AAZ adduct. After the addition of MZA, the CA/AAZ signal was no longer detectable. In its place, the peak of the CA/MZA adduct appeared very clearly (Fig. 8). A less intense peak attributable to the holo-hCA I adduct with both inhibitors was also revealed. Also, this behavior can be reasonably explained as described above, considering the particular reaction in the gas phase [63]. As expected, the stronger inhibitor causes the displacement of the weaker one, binding in turn to the protein.

In the opposite experiment, MZA was first incubated with hCA I, and then, AAZ was added. Both spectra showed only the signals due to the holo-CA and to the CA/MZA adduct (see SI), reflecting the tendency of MZA to preferentially bind the protein compared to the less strong inhibitor AAZ. Notably, no adduct with both inhibitors has been observed in this case.

### The case of auranofin

We finally compared the reactivity of the studied inhibitors and CA with a well-known system investigated in the previous works: the binding of the gold(I)–drug auranofin (AF) [38, 44, 80, 81]. A recent paper of ours has clearly demonstrated that AF binds selectively to the free and solvent-accessible cysteine residues of proteins [38]. In the presence of an accessible thiol, AF loses its thiosugar ligand and binds covalently through the gold(I) center directly to the sulfur atom of the thiol. Through our well-consolidated protocol for the ESI–MS analysis, adducts with AuPEt_3_^+^ fragment were observed for some representative proteins containing solvent-accessible free Cys residues; the exclusive binding on this amino acid residue has been clearly confirmed by competition studies with ebselen, an organoselenium compound that binds selectively and covalently to thiols [82–86].

**Fig. 9:**
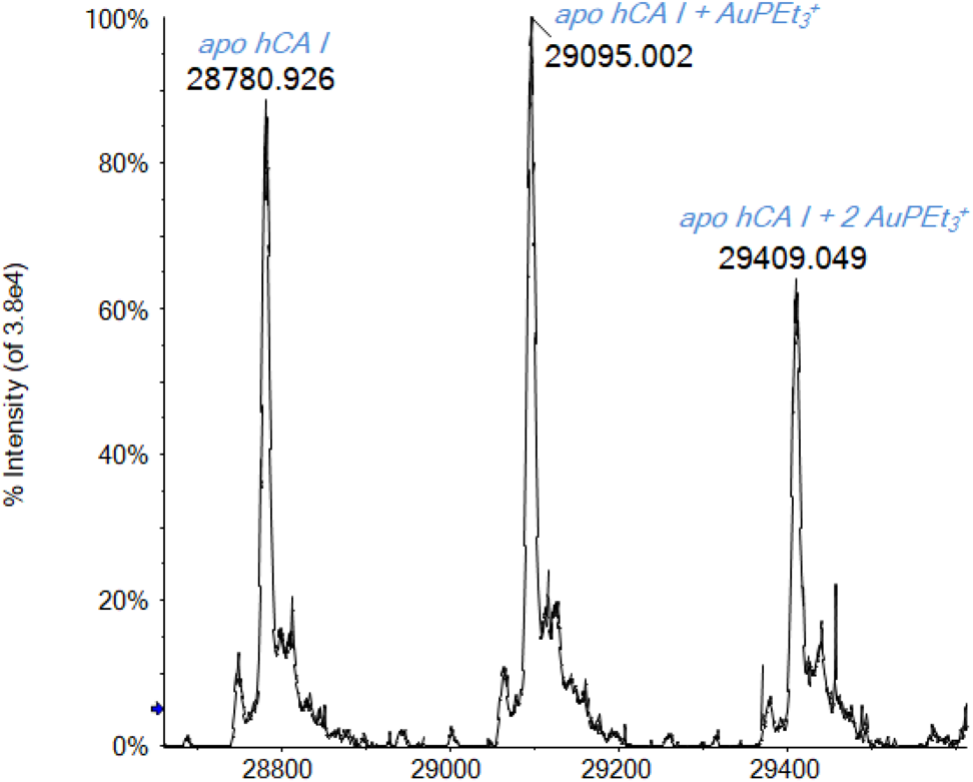
Deconvoluted ESI-Q-TOF mass spectrum of hCA I 7 × 10^–7^ M incubated for 5 min at 37 °C with AF (1:3 protein-to-metal ratio) in ammonium acetate solution 2 × 10^–3^ M (pH 6.8) and 0.1% v/v of formic acid

Especially for the case of CA I, the binding with AF takes place in acidic, denatured, conditions (0.1% v/v of formic acid, pH 2.9). The spectrum in Fig. 9 displays the deconvoluted mass spectrum of apo-CA with AF (1:3 protein to AF ratio) in the presence of acid. The signal of the apo protein is accompanied by two other peaks at 29,095 and 29,409 Da corresponding to the mono and bis adduct of the apo protein with AuPEt_3_^+^ fragment, respectively. The hCA I owns just one Cys residue (Cys212) not involved in disulphide bridges, therefore, potentially available for the binding with AF. However, the bis adduct most likely forms upon binding of two AuPEt_3_^+^ fragments to the thiol of Cys212, giving rise to the kinetically favored thiolated-bridged digold complex, as already observed by F. Shaw III [87].

**Fig. 10:**
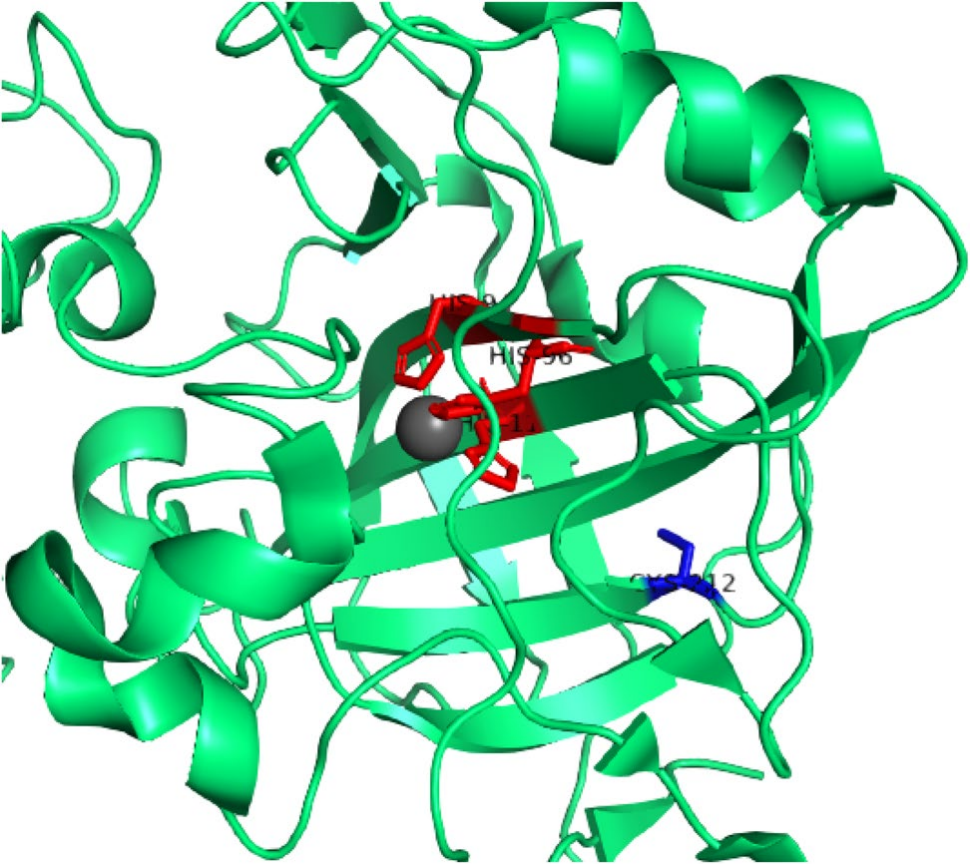
Ribbon representation of the hCA I structure (zoom on the catalytic site). The free cysteine side-chain is shown in blue, while the His residues in the active site are highlighted in red. From Protein Data Bank, entry 2NN7

Nevertheless, in the native-like conditions identified for hCA I, adduct formation has not been observed between AF and hCA I but only the holo-protein signal is detected (see SI). From this evidence, we inferred that, just after the partial unfolding of the protein, the free Cys residue becomes available for the binding to the gold center. Indeed, the Cys212 residue is not completely solvent-accessible in the CA I native state [88, 89], as it can be observed from the enzyme crystal structure reported in Fig. 10.

Only when some partial unfolding of the protein takes place (due for example to acidification), the Cys residue becomes spatially accessible for the binding with AF. Contrariwise, in the absence of a potential and accessible binding site, AF retains the thiosugar ligand and no interaction occurs.

Two additional experiments were conducted mixing both AF and MZA with CA in a solution of 1:3:3 protein/inhibitor/metallodrug ratio. First, we analyzed the sample in native-like conditions and just the mono-adduct between the holo-CA and MZA is detected (see SI). Again, the protein reacts completely with the inhibitor, but there is no AuPEt_3_^+^ biding. Adding to the same solution a 0.1% v/v of formic acid and repeating the ESI-MS analysis, the new mass spectrum is perfectly superimposable with the one displayed in Fig. 9 (see SI). In this case, the reveled adducts are only between AF and apo-CA; the Zn ion and the inhibitor are no more bound to the protein.

## Conclusions

Native mass spectrometry has been recognized as a rapid, sensitive, high throughput, and label-free method to directly investigate protein–ligand interactions, preserving all the biological functions of the macromolecule. However, it is worth reminding that the so-called “native conditions” are strictly related to the nature of the analyzed protein and to its chemico-physical properties [28]. In this paper, we investigated in depth the parameters that are involved in the ESI–MS analysis of human carbonic anhydrase, starting from the solution pH value up to the critical instrumental parameters. We found that the best conditions for preserving the CA tertiary structure, jointly to its biological activity, require a solution pH value near to neutrality; therefore, the conventional addition of formic acid must be avoided. In fact, the resulting acidic conditions cause the release of the Zn ion and prevent the possibility to bind the inhibitor.

Once found the best conditions for CA analysis, we extended the MS study to the protein binding of three sulfonamide and one dithiocarbamate inhibitors. In studying these protein/inhibitor adducts, the declustering potential turned out to be another fundamental parameter to be considered to avoid an artificial alteration of the formed adduct.

Then, through this powerful and reliable methodology, we proved that the four inhibitors react rapidly and almost completely with the protein, forming mono and bis adducts in the case of sulfonamides and only mono adducts with dithiocarbamate; the adducts were characterized in detail.

As completion of this study, some competition experiments between inhibitors with different affinity for CA were performed. Also, in this case, the technique turned out to be extremely reliable, highlighting a good correlation between adduct formation and the relative inhibitors’ affinities for the protein itself.

Finally, some further experiments were carried out with the gold drug auranofin. This latter compound can be considered as a site-specific ligand, reacting only with free and solvent-accessible cysteine residues. Although auranofin does not hinder at all the reaction of CA with its inhibitors, it reacts only when the protein is partially unfolded, thus making the free cysteine residue accessible. At the same time, with a partial loss of the protein tertiary structure, the Zn ion and the inhibitor are released once again.

## Electronic supplementary material

Below is the link to the electronic supplementary material.

Supplementary file1 (PDF 1014 kb)
