# Supplementary material for: Native mass spectrometry of human carbonic anhydrase I and its inhibitor complexes
Source: J Biol Inorg Chem. 2020 Sep 14;25(7):979–93. doi: 10.1007/s00775-020-01818-8 (PMC7584553; doi:10.1007/s00775-020-01818-8)
Supplement: Supplementary file 1 — Supplementary file1 (PDF 1014 kb) [file 775_2020_1818_MOESM1_ESM.pdf]

Supplementary Material

Native mass spectrometry of human carbonic anhydrase I and its inhibitor complexes

Carlotta Zoppi,[1] Alessio Nocentini,[2] Claudiu T. Supuran,[2] Alessandro Pratesi,[,3] Luigi Messori*[,1]*

[1] Laboratory of Metals in Medicine (MetMed), Department of Chemistry "Ugo Schiff", University of Florence, Via della Lastruccia 3-13, 50019 Sesto Fiorentino, Italy.
[2] Department of Neurofarba, University of Florence, Section of Pharmaceutical and Nutraceutical Sciences, Via U. Schiff 6, 50019 Sesto Fiorentino, Italy
[3] Department of Chemistry and Industrial Chemistry, University of Pisa, Via G. Moruzzi 13, 56124 Pisa, Italy.

Corresponding authors:
Alessandro Pratesi E-mail: alessandro.pratesi@unipi.it
Luigi Messori, E-mail: luigi.messori@unifi.it

hCA I 2
SLC-0111 2
MZA 4
AAZ 5
DTC 6
AF 7
MZA and AF competition experiment 7
MZA and AAZ competition experiment 8
AAZ and MZA competition experiment 9

hCA I

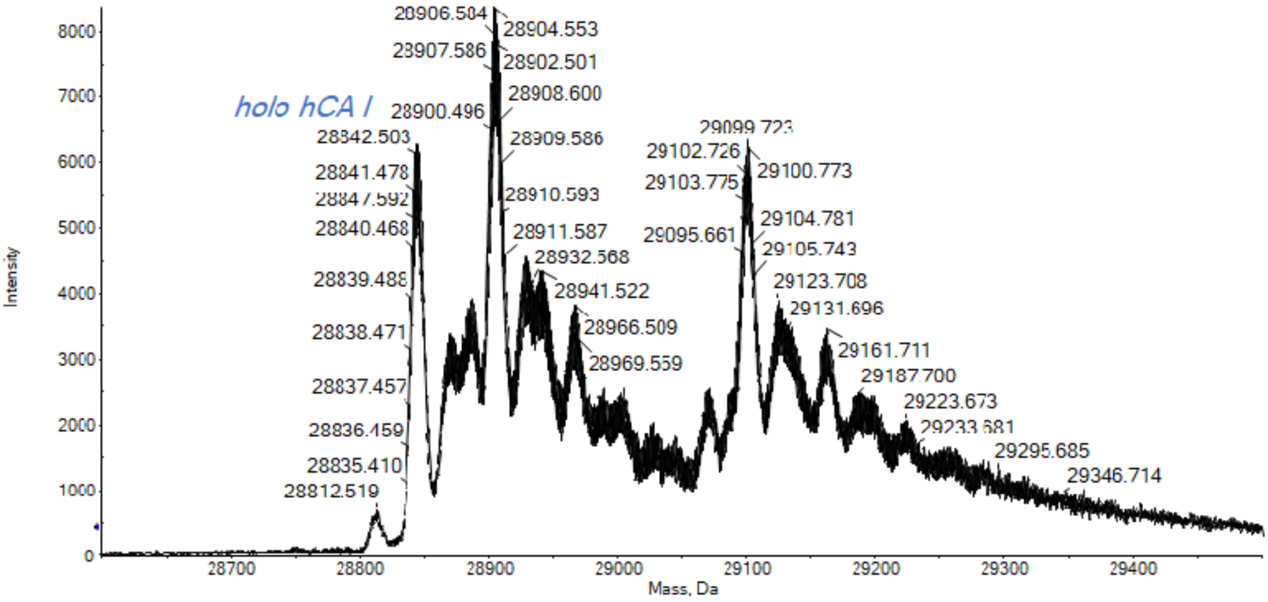

Figure 1 Deconvoluted ESI-Q-TOF mass spectrum of hCA I 7x10^{-7} M in ammonium acetate solution 2x10^{-3} M (pH 6.8) DP 100 V

SLC-0111

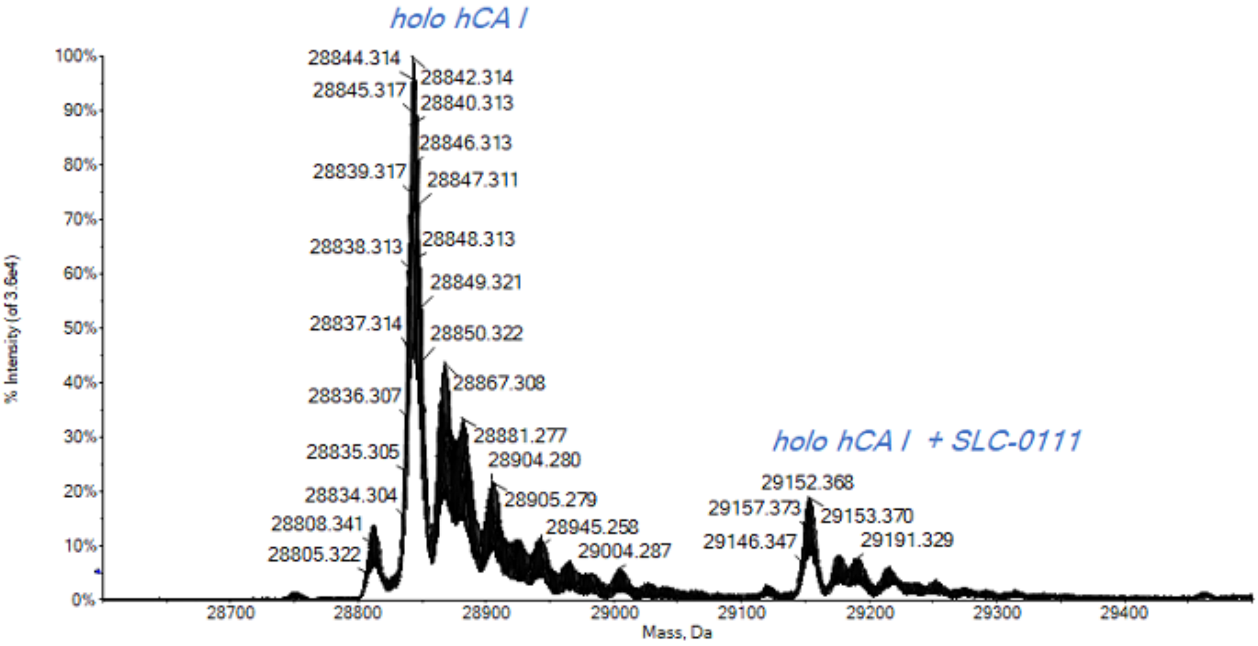

Figure 2 Deconvoluted ESI Q TOF spectrum of hCA I solution 7x10-7 M with SLC-0111 (1:1 protein/inhibitor ratio) in AmAc buffer 2 mM (pH 6.8), DP 300 V

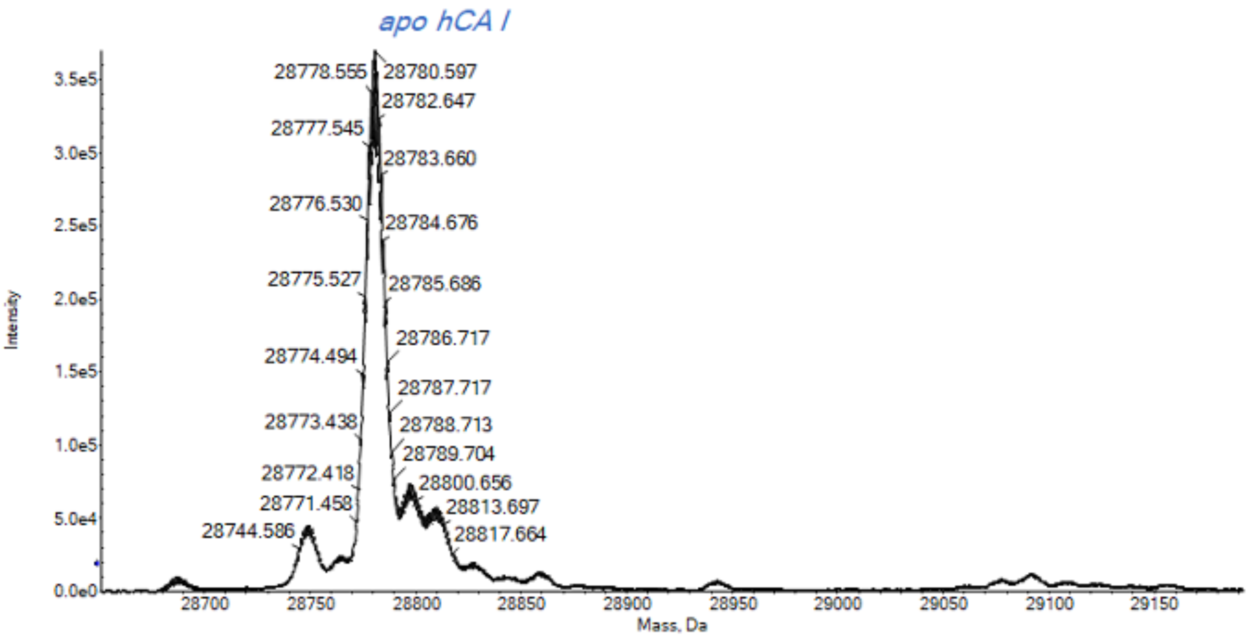

Figure 3 Deconvoluted ESI Q TOF spectrum of hCA I solution 7x10-7 M with SLC-0111 (1:1 protein/inhibitor ratio) in AmAc buffer 2 mM (pH 6.8) 0.1% v/v of formic acid (FoA), DP 300 V

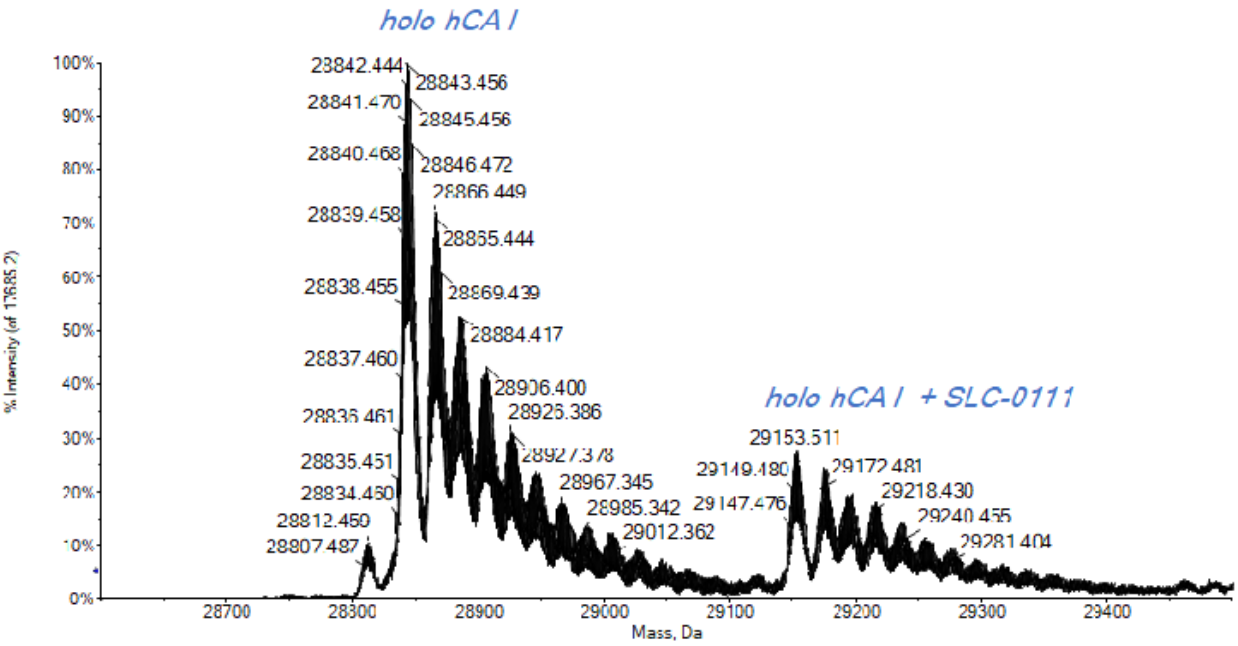

Figure 4 Deconvoluted ESI Q TOF spectrum of hCA I solution 7x10-7 M with SLC-0111 (1:10 protein/inhibitor ratio) in AmAc buffer 2 mM (pH 6.8), DP 300 V

MZA

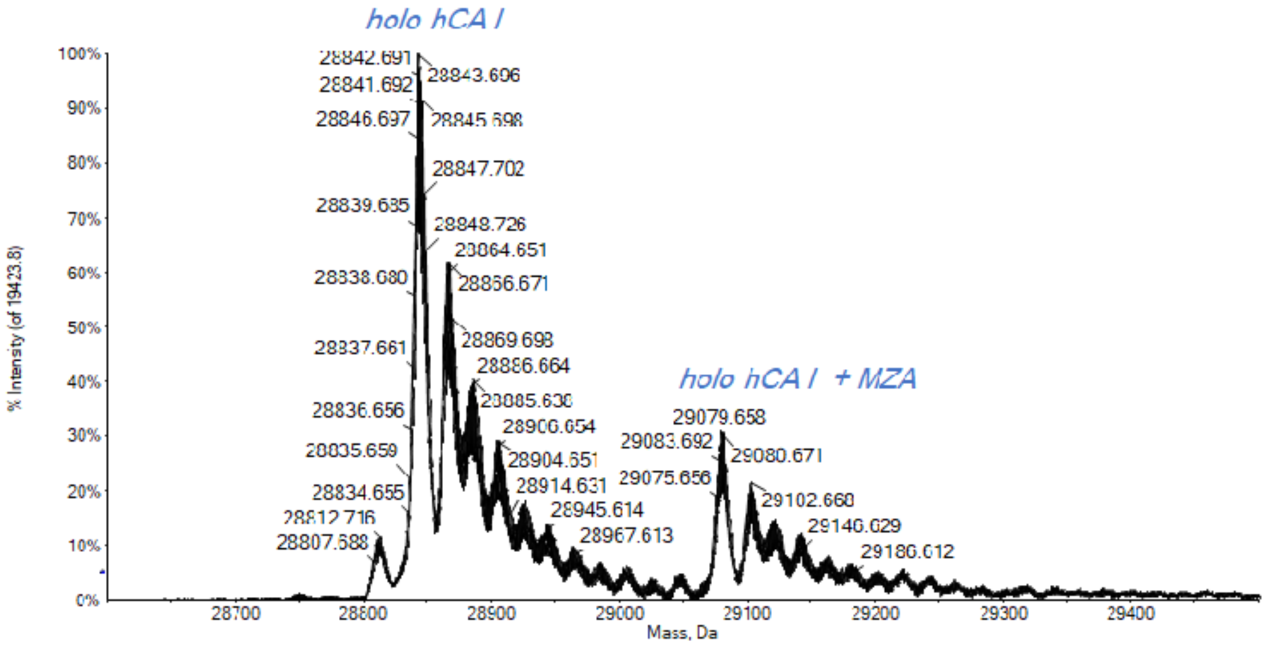

Figure 5 Deconvoluted ESI Q TOF spectrum of hCA I solution 7x10-7 M with MZA (1:1 protein/inhibitor ratio) in AmAc buffer 2 mM (pH 6.8), DP 300 V

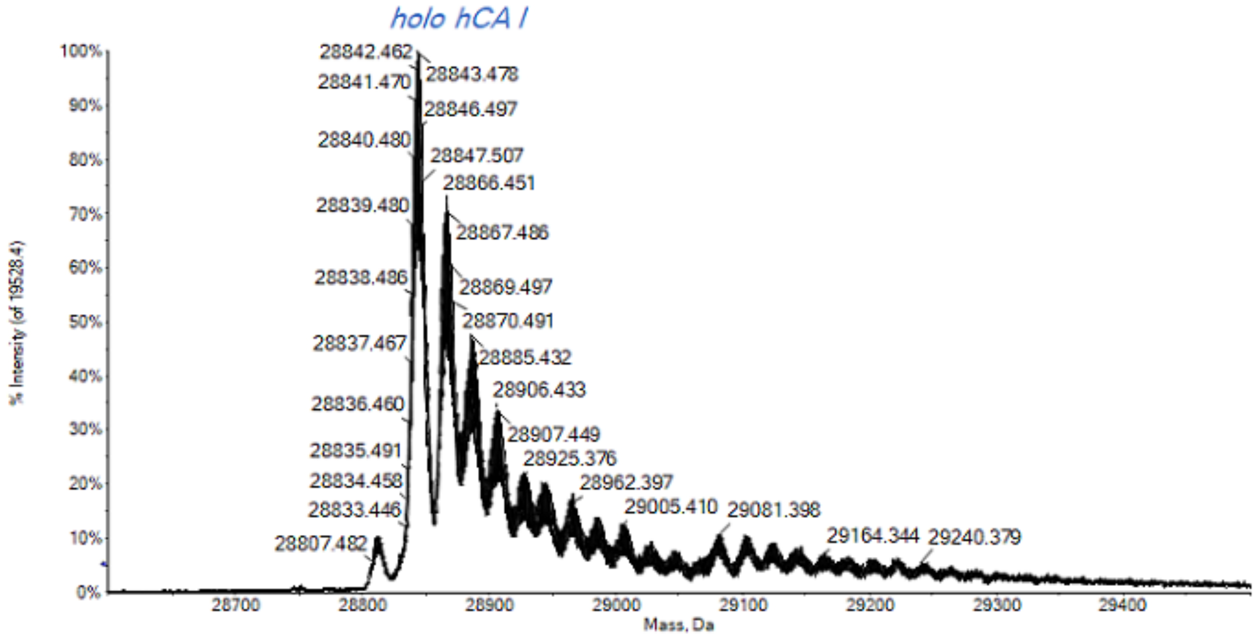

Figure 6 Deconvoluted ESI Q TOF spectrum of hCA I solution 7x10-7 M with MZA (1:10 protein/inhibitor ratio) in AmAc buffer 2 mM (pH 6.8), DP 300 V

AAZ

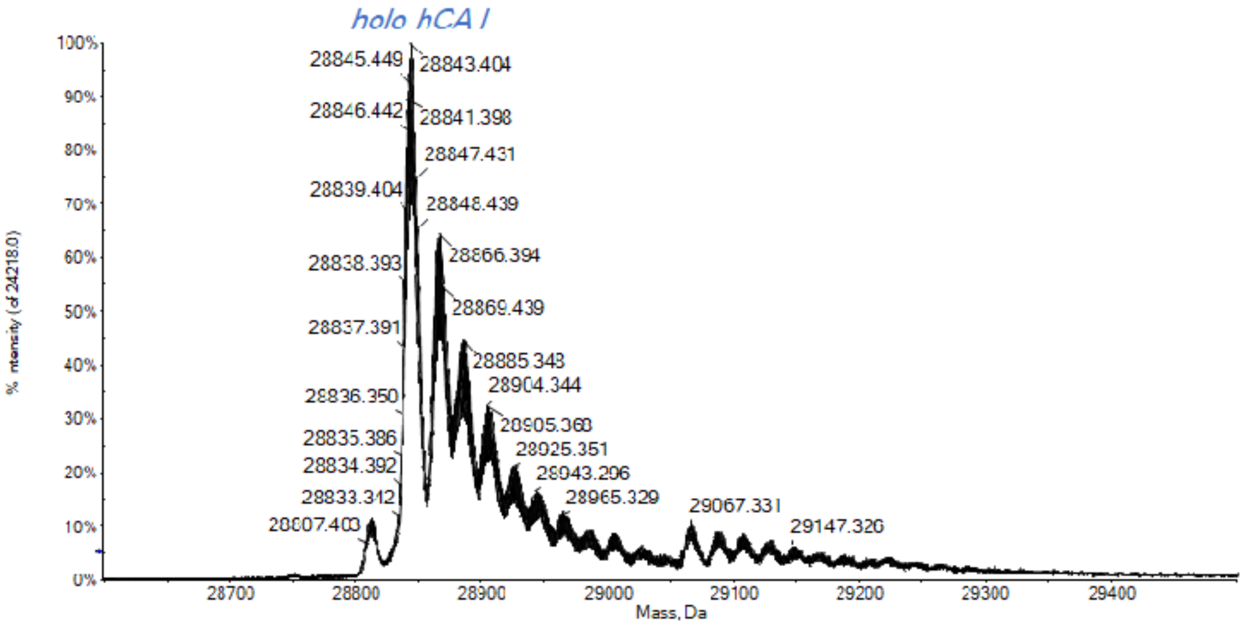

Figure 7 Deconvoluted ESI Q TOF spectrum of hCA I solution 7x10-7 M with AAZ (1:1 protein/inhibitor ratio) in AmAc buffer 2 mM (pH 6.8), DP 300 V

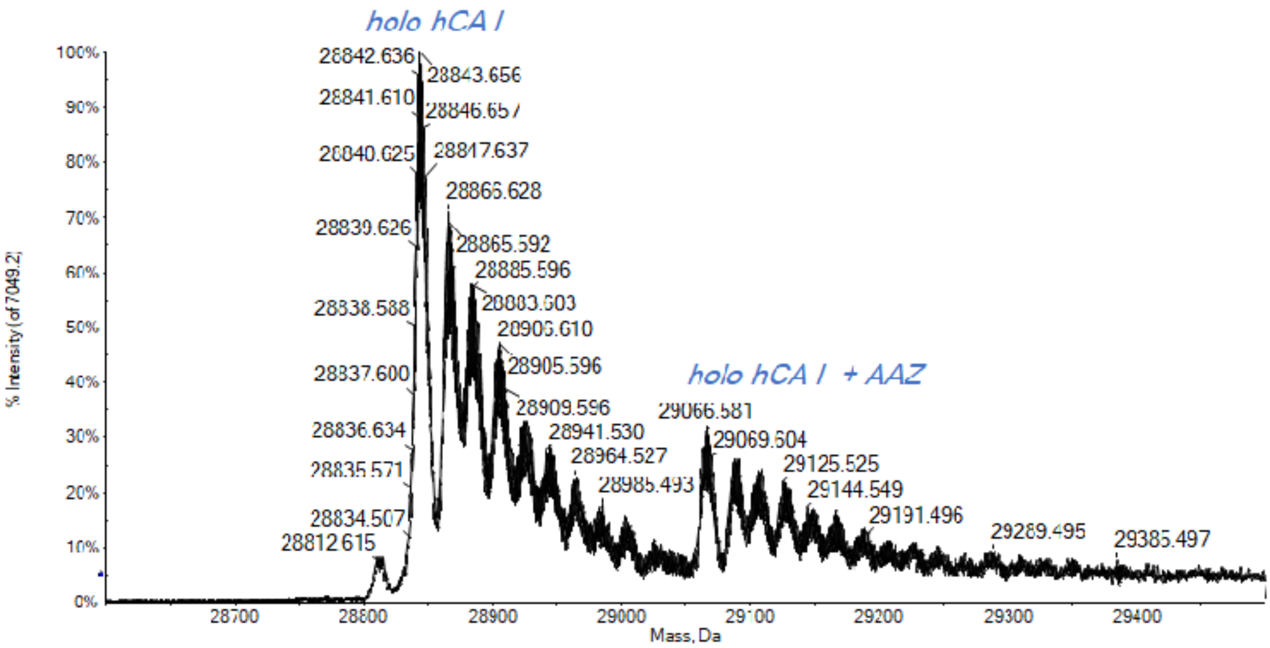

Figure 8 Deconvoluted ESI Q TOF spectrum of hCA I solution 7x10-7 M with AAZ (1:10 protein/inhibitor ratio) in AmAc buffer 2 mM (pH 6.8), DP 300 V

DTC

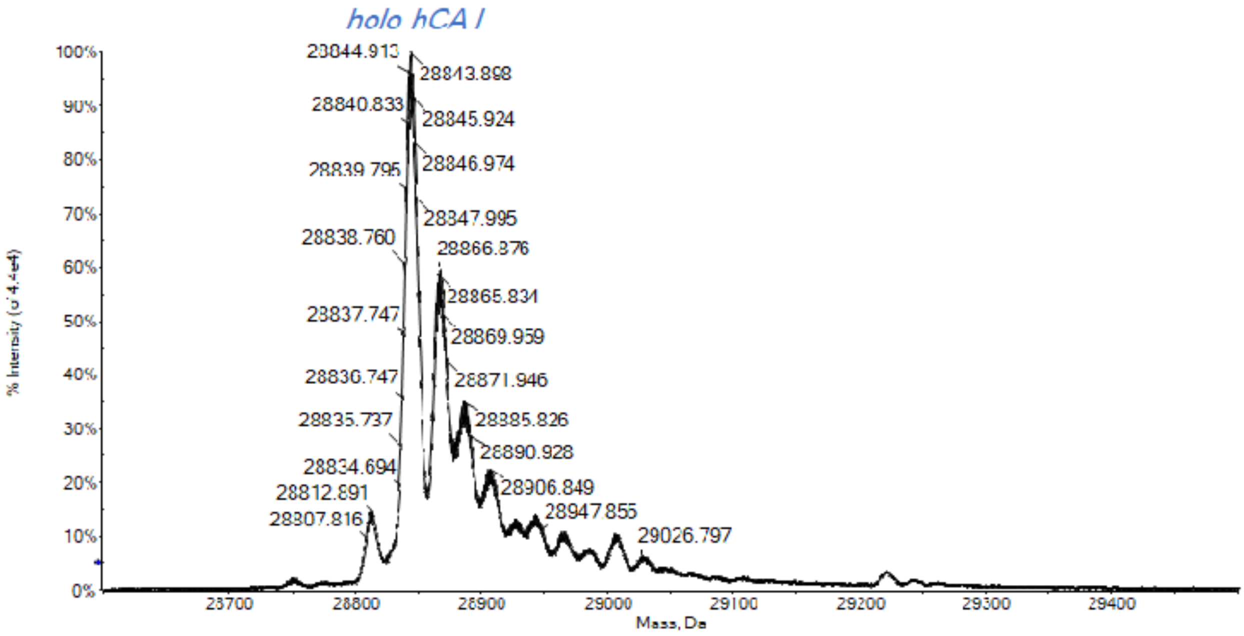

Figure 9 Deconvoluted ESI Q TOF spectrum of hCA I solution 7x10-7 M with DTC (1:1 protein/inhibitor ratio) in AmAc buffer 2 mM (pH 6.8), DP 300 V

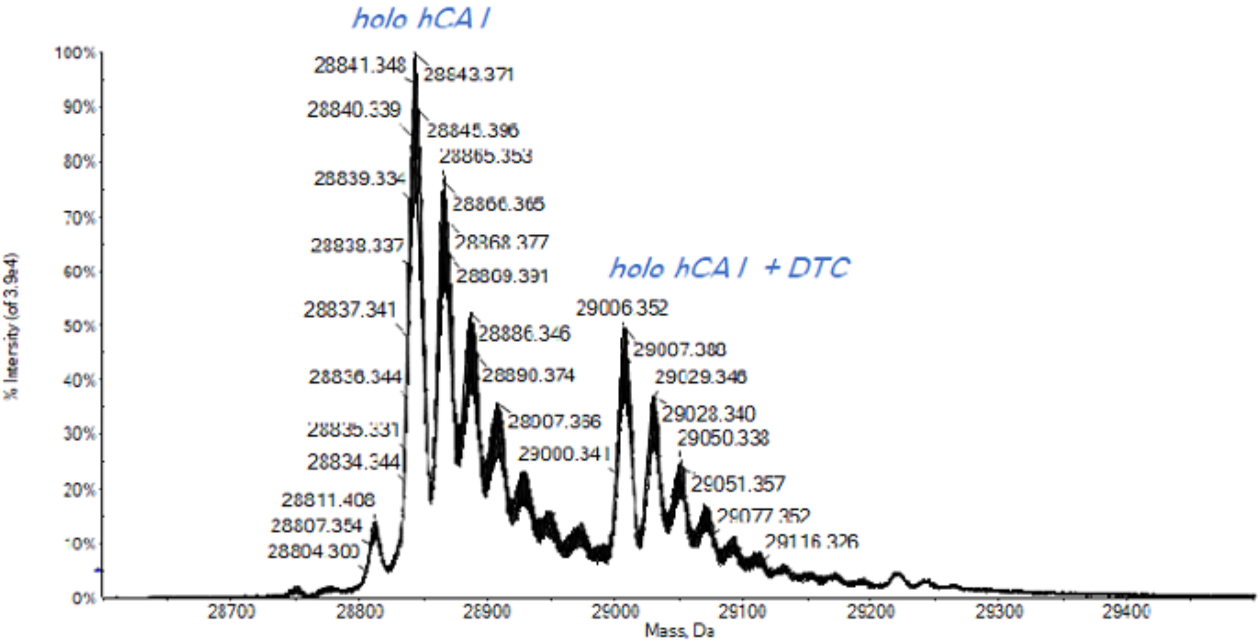

Figure 10 Deconvoluted ESI Q TOF spectrum of hCA I solution 7x10-7 M with DTC (1:10 protein/inhibitor ratio) in AmAc buffer 2 mM (pH 6.8), DP 300 V

AF

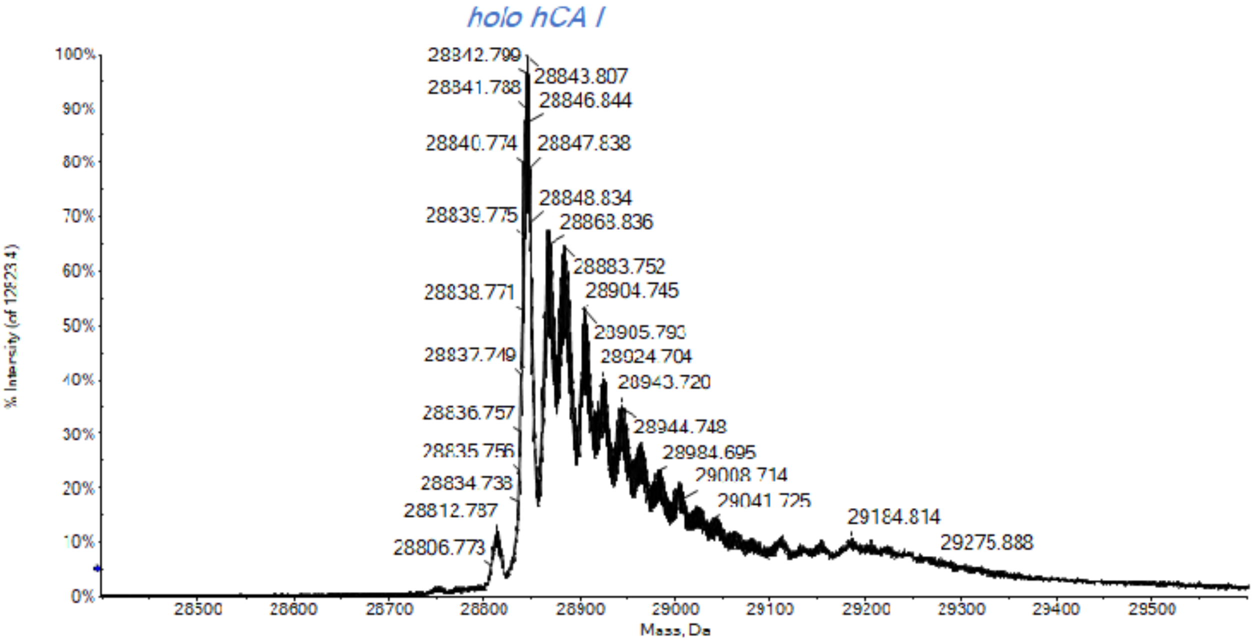

Figure 11 Deconvoluted ESI Q TOF spectrum of hCA I solution 7x10-7 M with AF (1:3 protein to metal ratio) in AmAc buffer 2 mM (pH 6.8)

MZA and AF competition experiment

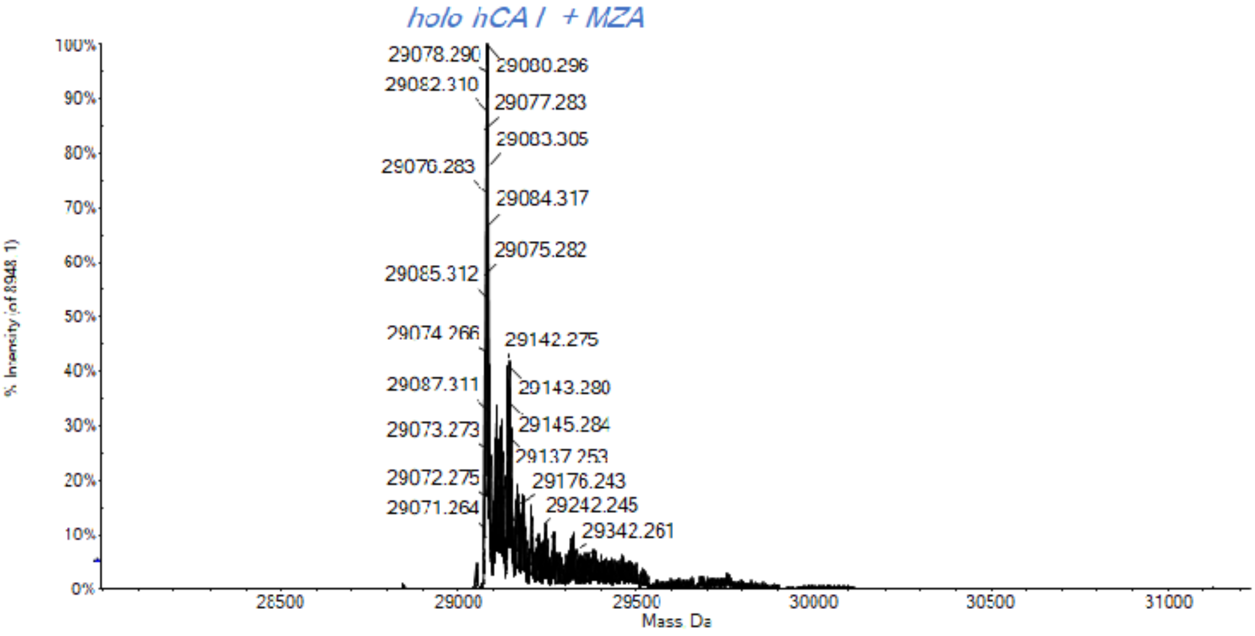

Figure 12 Deconvoluted ESI Q TOF spectrum of hCA I solution 7x10-7 M with AF and MZA (1:3:3 protein to metal to inhibitor ratio) in AmAc buffer 2 mM (pH 6.8) , DP 200 V

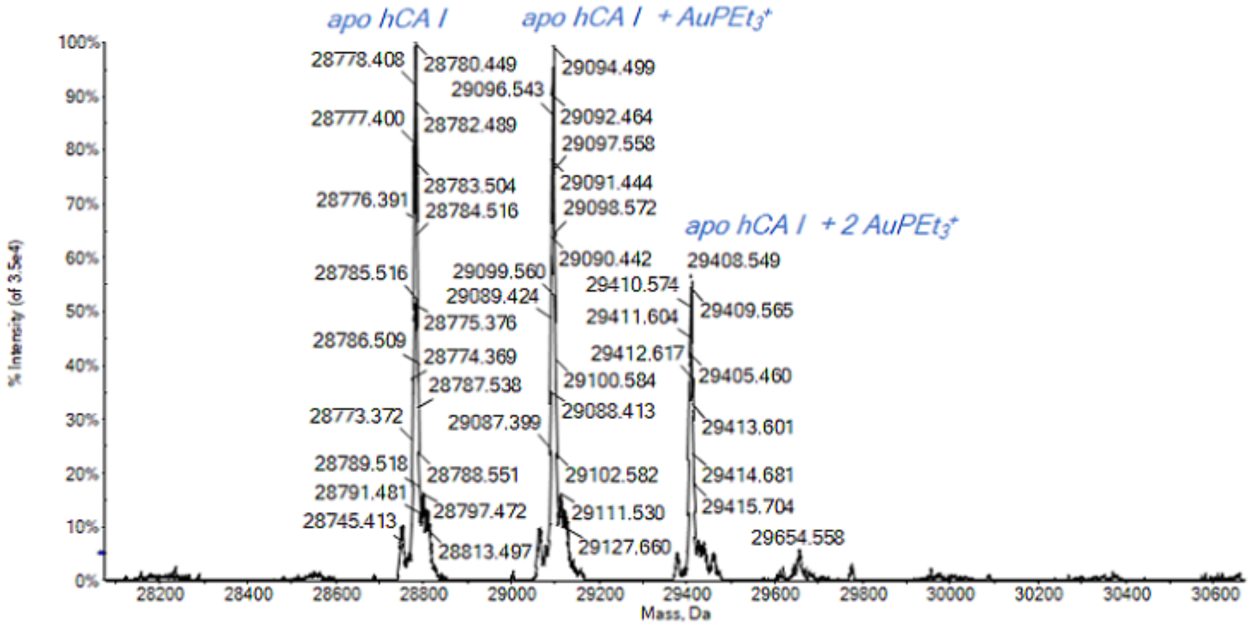

Figure 13 Deconvoluted ESI Q TOF spectrum of hCA I solution 7x10-7 M with AF and MZA (1:3:3 protein to metal to inhibitor ratio) in AmAc buffer 2 mM (pH 6.8), *0.1% FoA v/v*

MZA and AAZ competition experiment

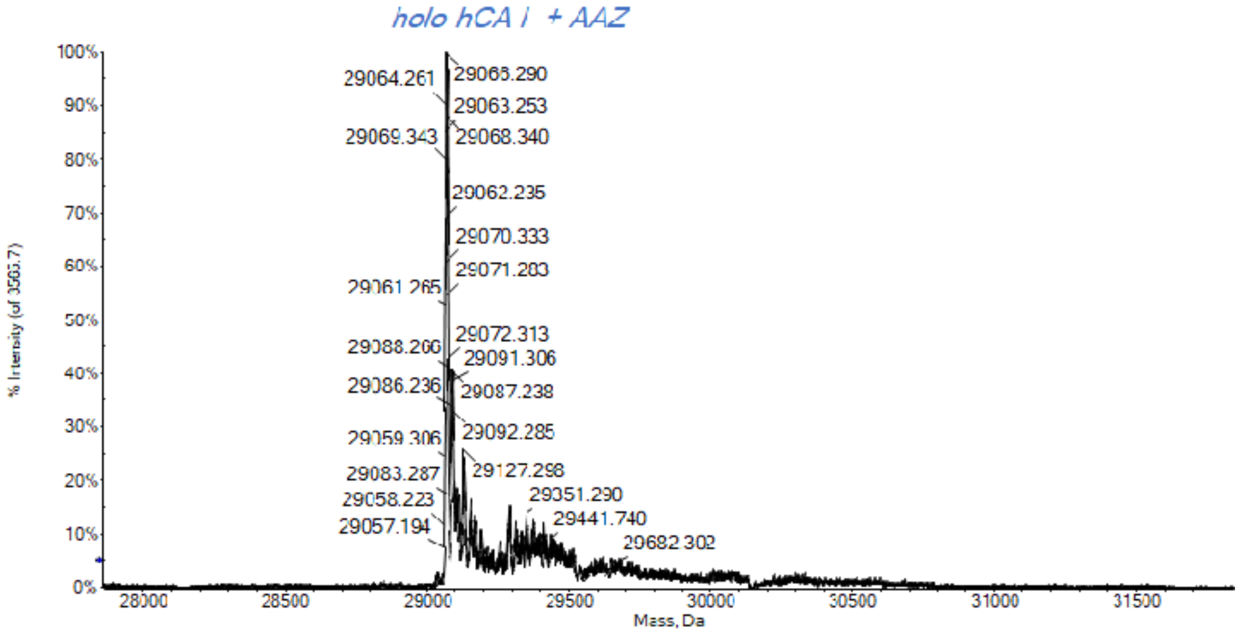

Figure 14 Deconvoluted ESI Q TOF spectrum of hCA I solution 7x10-7 M with AAZ (1:10 protein/inhibitor ratio) in AmAc buffer 2 mM (pH 6.8), DP 200 V

AAZ and MZA competition experiment

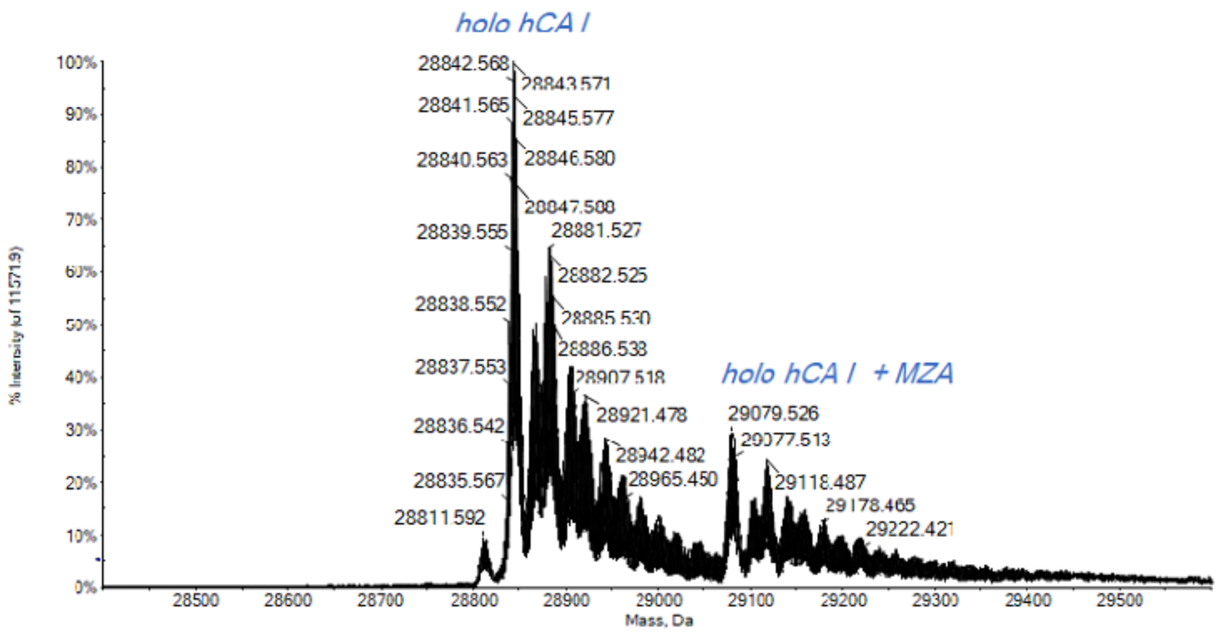

Figure 15 Deconvoluted ESI Q TOF spectrum of hCA I solution 7x10-7 M with MZA (1:10 protein/inhibitor ratio) in AmAc buffer 2 mM (pH 6.8), DP 300 V

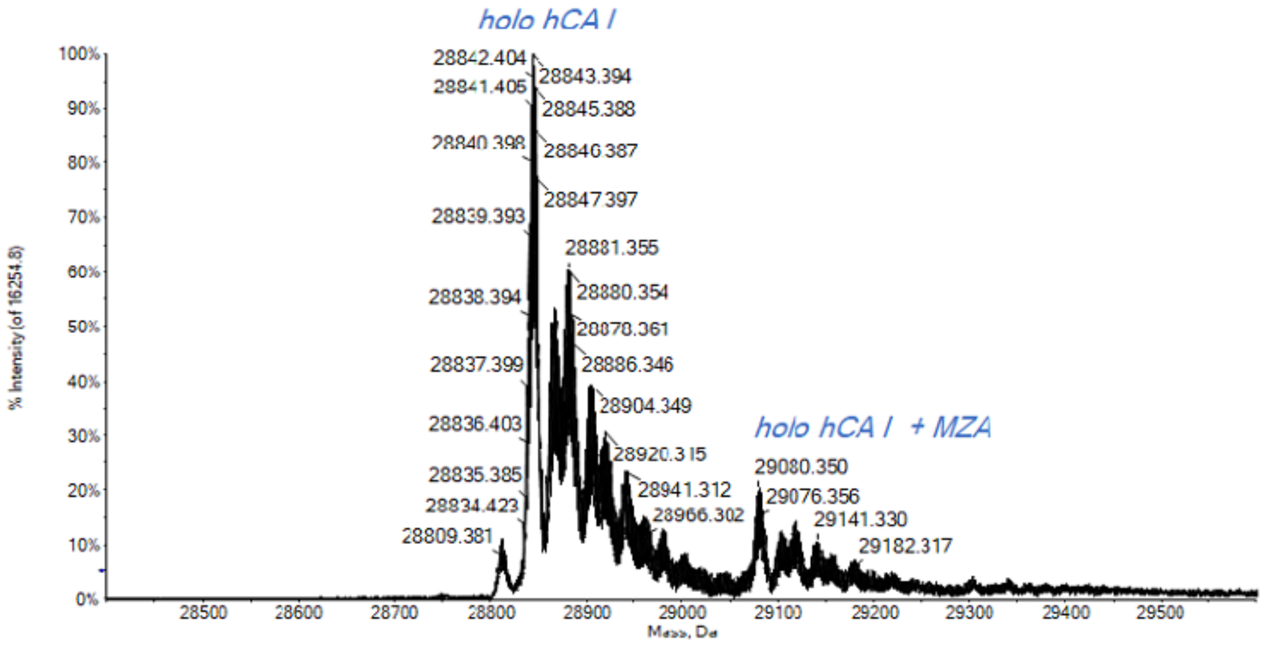

Figure 16 Deconvoluted ESI Q TOF spectrum of hCA I solution 7x10-7 M with MZA and AAZ (1:10:10 protein/inhibitor 1/ inhibitor 2 ratio) in AmAc buffer 2 mM (pH 6.8), DP 300 V
